# New approach to determine the surface and interface thermodynamic properties of H-β-zeolite/rhodium catalysts by inverse gas chromatography at infinite dilution

**DOI:** 10.1038/s41598-020-78071-1

**Published:** 2020-12-01

**Authors:** Tayssir Hamieh, Ali Ali Ahmad, Thibault Roques-Carmes, Joumana Toufaily

**Affiliations:** 1 SATIE-IFSTTAR, University Gustave Eiffel, Campus de Marne-La-Vallée, 25, allée des Marronniers, 78000 Versailles, France; 2 Laboratory of Materials, Catalysis, Environment and Analytical Methods (MCEMA), Faculty of Sciences and EDST, Lebanese University, Hariri Campus, Hadath, Beirut Lebanon; 3 LEADDER Laboratory, Faculty of Sciences and EDST, Lebanese University, Hariri Campus, Hadath, Beirut Lebanon; 4 Laboratoire Réactions Et Génie Des Procédés (LRGP), UMR CNRS, 7274, Université de Lorraine, 1 Rue Grandville, 54001 Nancy, France

**Keywords:** Chemistry, Engineering, Materials science

## Abstract

The thermodynamic surface properties and Lewis acid–base constants of H-β-zeolite supported rhodium catalysts were determined by using the inverse gas chromatography technique at infinite dilution. The effect of the temperature and the rhodium percentage supported by zeolite on the acid base properties in Lewis terms of the various catalysts were studied. The dispersive component of the surface energy of Rh/H-β-zeolite was calculated by using both the Dorris and Gray method and the straight-line method. We highlighted the role of the surface areas of n-alkanes on the determination of the surface energy of catalysts. To this aim various molecular models of n-alkanes were tested, namely Kiselev, cylindrical, Van der Waals, Redlich–Kwong, geometric and spherical models. An important deviation in the values of the dispersive component of the surface energy ${\gamma }_{s}^{d}$ determined by the classical and new methods was emphasized. A non-linear dependency of ${\gamma }_{s}^{d}$ with the specific surface area of catalysts was highlighted showing a local maximum at 1%Rh. The study of *RTlnVn* and the specific free energy *∆G*^*sp*^(*T*) of n-alkanes and polar solvents adsorbed on the various catalysts revealed the important change in the acid properties of catalysts with both the temperature and the rhodium percentage. The results proved strong amphoteric behavior of all catalysts of the rhodium supported by H-β-zeolite that actively react with the amphoteric solvents (methanol, acetone, tri-CE and tetra-CE), acid (chloroform) and base (ether) molecules. It was shown that the Guttmann method generally used to determine the acid base constants *K*_*A*_ and *K*_*D*_ revealed some irregularities with a linear regression coefficient not very satisfactory. The accurate determination of the acid–base constants *K*_*A*_*, K*_*D*_ and *K* of the various catalysts was obtained by applying Hamieh’s model (linear regression coefficients approaching *r*^2^ ≈ 1.000). It was proved that all acid base constants determined by this model strongly depends on the rhodium percentage and the specific surface area of the catalysts.

## Introduction

The determination of the surface and interface properties of solid materials is of vital importance in many industrial domains including catalysis, biomedicine, chemical engineering, adsorption, adhesion, membrane fabrication, polymers and composites, clays^1,2^, nanomaterials and clay-polymer composites^3,4^, pharmaceutical and food products^5–8^. One of the most popular and interesting technique to determine the surface properties of solid materials is the inverse gas chromatography (IGC) at infinite dilution. The IGC technique can advantageously give access to the acid base properties in Lewis terms as well as to the thermodynamic parameters such as specific free energy, enthalpy and entropy of adsorption. In addition, Lewis acid–base character of the surface, surface nanoroughness parameter, can be also determined^5–12^. The IGC technique appears a real source of physiochemical data of surfaces and interfaces^13^ allowing the observation of the interactions between oxides, polymers or polymers adsorbed on oxides and organic solvent systems^14^. This is an important tool, precise, sensitive, and more competitive to determine the heterogeneous surfaces of textiles, their physicochemical properties^15^, and to determine surface energy and surface area of powdered materials^16,17^. In previous studies^18,19^, we used IGC technique to determine the surface characteristics of various oxides and polymers or polymers adsorbed on oxides, especially, their surface energies, their interactions with some organic molecules and the acid–base properties of solid materials or nanomaterials. The IGC technique was preferentially applied to characterize the surface properties of catalysts or metals containing catalysts that can be advantageously used in industrial applications^20–22^.

It is well known that rhodium is used in automobile industries during the manufacturing of automobile catalytic converts^20^. It plays an important role in the oxidation of ammonia and carbon monoxide and also in the elimination of nitric oxide^21,22^. On the other hand, beta zeolite was proved to be an excellent catalyst due to the relatively high density of Brønsted acid sites and favorable pore structure^23–25^. Zeolite can be considered as an interesting support for metal catalysts. Moloy et al.^26^ studied the adsorption properties of zeolite and metal loaded zeolite. However, they did not provide details on the specific surface properties, the acid base constants in Lewis terms and the surface energy of H-β-zeolite supported rhodium catalysts.

In this paper, a new approach for the determination of the surface and interfacial properties of H-β-zeolite and the rhodium impregnated in H-β-zeolite catalysts is developed. We used the inverse gas chromatography technique at infinite dilution, Papirer’s approach^27–29^ and Hamieh’s model^18,19^ to determine the specific free enthalpy and enthalpy of adsorption and the acid–base constants of the above materials. The dispersive component of the surface energy of such catalysts was also studied by using the various molecular models of n-alkanes.

## Theory and methods

Inverse gas chromatography can be considered as powerful technique used to determine the superficial phenomena, the surface energy, the specific free energy enthalpy and entropy of adsorption and the acid–base properties of solid materials. IGC technique was applied in this study to determine the changes of the superficial properties of H-β-zeolite/rhodium catalysts as a function of the temperature. Probes of known properties were injected into the column containing the solid. The retention times of these probes, measured at infinite dilution, allowed us to determine the interactions between model organic molecules and the solid assuming that there was no interaction between the probe molecules.

In parallel, the surface specific area of the various catalyst samples was determined by using Brunauer–Emmett–Teller (BET). The nitrogen adsorption–desorption experiments were carried out using BET gas adsorption method at 77 K, in an automatic Micromeritics ASAP 2420 apparatus. The samples were degassed under vacuum for 2 h at 100 °C followed by 300 °C for 10 h before the measurements. The specific surface *S*_*BET*_ area was determined by using the classical BET method. The mesopore size distribution of the catalysts were calculated using the model of Barrett–Joyner–Halenda (BJH).

### Retention volume

The net retention volume *Vn* was calculated from:

1 $$ Vn \, = \, j \, D_{c} \left( {t_{R} - \, t_{0} } \right) $$

where *t*_*R*_ is the retention time of the probe, *t*_*0*_ the zero retention reference time measured with a non adsorbing probe such as methane, *D*_*c*_ the corrected flow rate and *j* a correction factor taking into account the compression of the gas^30^.

*D*_*c*_ and *j* are respectively given by the following expressions:

2 $${D}_{c}={D}_{m}\frac{{T}_{c}}{{T}_{a}} \frac{\eta \left({T}_{c}\right)}{\eta \left({T}_{a}\right)}$$

and

3 $$j=\frac{3}{2} \frac{{\left(\frac{\Delta P+ {P}_{0}}{{P}_{0}}\right)}^{2}-1}{{\left(\frac{\Delta P+ {P}_{0}}{{P}_{0}}\right)}^{3}-1}$$

where *D*_*m*_ is the measured flow rate, *T*_*c*_ the column temperature, *T*_*a*_ the room temperature, *η(T) *the gas viscosity at temperature *T*, *P*_*0*_ the atmospheric pressure and *∆P* the pressure variation.

### Determination of the dispersive component of the surface energy of a solid

The free enthalpy of adsorption *∆G*^*0*^ of n-alkanes on a solid is given by:

4 $$ \Delta G^{0} = \, - \, RT \, ln \, Vn \, + \, C $$

where *R* is the ideal gas constant, *T* the absolute temperature and *C* a constant depending on the reference state of adsorption. In the case of n-alkanes, *∆G*^*0*^ is equal to the free energy of adsorption corresponding to dispersive interactions *∆G*^*d*^ only.

#### The increment method

Dorris and Gray^31^ proposed the increment method by applying the well-known relationship of Fowkes^32^ which gives at the same time the dispersive component of the surface energy of solids ${\gamma }_{s}^{d}$ by using the geometric mean of the dispersive components (exponent *d)* of the surface energy of the probe ${\gamma }_{l}^{d}$ and the solid ${\gamma }_{s}^{d}$:

5 $$ W_{a} = 2 \sqrt {\gamma_{l}^{d} \gamma_{s}^{d} } $$

where *W*_*a*_ is the work of adhesion between the probe and the solid.

This energy of adhesion can be correlated to the free enthalpy of adsorption following

6 $$ \Delta G^{0} = {\mathcal{N}},aW_{a} = 2{\mathcal{N}}a \sqrt {\gamma_{l}^{d} \gamma_{s}^{d} } $$

where $\mathcal{N}$ is Avogadro’s number and *a* the surface area of o adsorbed molecule on the solid.

Dorris and Gray were the first who determined the dispersive component of the surface energy of a solid by considering the increment of $\Delta {G}_{-CH2-}^{0}$ per methylene group in the n-alkanes series of general formula *C*_*n*_*H*_*2(n*+*1)*_. They defined the increment $\Delta {G}_{-CH2-}^{0}$ by:

7 $$\Delta {G}_{-CH2-}^{0}= \Delta {G}^{0}\left({C}_{n+1}{H}_{2(n+2)}\right)-\Delta {G}^{0}\left({C}_{n}{H}_{2(n+1)}\right)$$

where ${C}_{n}{H}_{2(n+1)}$ and ${C}_{n}{H}_{2(n+1)}$ represent the general formula of two consecutive n-alkanes.

By using the retention volumes ${V}_{n}\left({C}_{n}{H}_{2(n+1)}\right)$ and ${V}_{n}\left({C}_{n+1}{H}_{2(n+2)}\right)$ of two consecutive n-alkanes and the relation (4), the dispersive component of the surface energy ${\gamma }_{s}^{d}$ can be determined by the following equation:

8 $${\gamma }_{s}^{d}=\frac{{\left[RTln\left[\frac{{V}_{n}\left({C}_{n+1}{H}_{2(n+2)}\right)}{{V}_{n}\left({C}_{n}{H}_{2(n+1)}\right)}\right]\right]}^{2}}{4{\mathcal{N}}^{2 }{a}_{-CH2- }^{2}{\gamma }_{-CH2-}}$$

where *a*_*-CH2-*_ is the surface area of methylene group (*a*_*-CH2-*_ = 6 Å^2^) and ${\gamma }_{-CH2-}$ the surface energy of –CH_2_– group of a polyethylene polymer (with a finite molecular mass). The latter is given by:

9 $$ \gamma_{ - CH2 - } = { 52}.{6}0{3 }{-} \, 0.0{58}T ({\text{T in K}};\gamma_{ - CH2 - } {\text{in mJ}}/{\text{m}}^{{2}} ) $$

By applying Dorris and Gray’s method, we determined the dispersive cponent of the surface energy ${\gamma }_{s}^{d}$ of H-β-zeolite for various temperatures. We only gave here the value determined at 480 K which was equal to ${\gamma }_{s}^{d}$ = 240.3 mJ/m^2^. The variation of ${\gamma }_{s}^{d}$(*T*) of H-β-zeolite as a function of the temperature is given by the following straight-line equation:

10 $$ \gamma_{s}^{d} (T) = { 327 } - 0.{\text{183 T}}.{66} $$

Note that the temperature T is in K while ${\gamma }_{s}^{d}$ is expressed in mJ/m^2^. The correlation coefficient was R^2^ = 0.9994.

#### The n-alkane straight-line method

This method, also based on Fowkes approach^32^, replaced the free enthalpy of adsorption by its value taken from relation (4). It leads to the following relationship:

11 $$ RT \, ln \, Vn \, + \, C = 2{\mathcal{N}}a \sqrt {\gamma_{l}^{d} \gamma_{s}^{d} } $$

By plotting *RTlnVn* as a function of $2\mathcal{N}a \sqrt{{\gamma }_{l}^{d}}$ of n-alkanes, one obtains a typical straight line that allows to deduce, from its slope, the value of dispersive component ${\gamma }_{s}^{d}$ of the surface energy of the solid.

**Figure 1:**
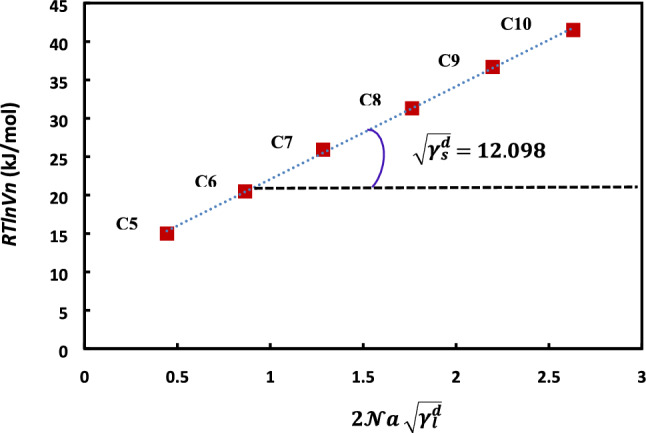
Variations of the retention volume of n-alkanes (from n-pentane C5 to n-decane C10) versus $2\mathcal{N}a \sqrt{{\gamma }_{l}^{d}}$ of probes of H-β-zeolite*.*

The evolution of *RTlnVn* as a function of $2\mathcal{N}a \sqrt{{\gamma }_{l}^{d}}$ of n-alkanes adsorbed on H-β-zeolite is reproduced in the Fig. 1. The experimental relation can be extracted:

12 $$RTlnVn=12.098 \left(2\mathcal{N}a \sqrt{{\gamma }_{l}^{d}}\right)+9.962;r2=0.9992$$

The slope of the straight line is $\sqrt{{\gamma }_{s}^{d}}=12.098$ and then ${\gamma }_{s}^{d}$ = 146.36 mJ/m^2^.

**Figure 2:**
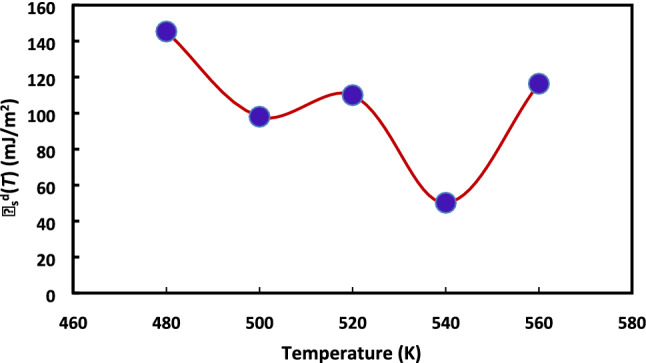
Variations of the dispersive component ${\gamma }_{s}^{d}$(*T*) of the surface energy of H-β-zeolite versus the temperature T.

The same method was applied, at different temperatures, in order to obtain the values ${\gamma }_{s}^{d}$(*T*) of H-β-zeolite at different temperatures. The results are displayed in the Fig. 2.

A non-linear variation of ${\gamma }_{s}^{d}$(*T*) with the temperature can be noticed. This is certainly due to the presence of hydroxide layer on the zeolite surface which is likely to evolve with the heat treatment. In addition, a large deviation between the results obtained by this method compared to those of Dorris and Gray’s method is observed. This is because of the high temperatures reaching 560 K that can extremely affect the values of the surface tensions of n-alkanes depending on the temperature interval; whereas, the surface tension of ethylene group is given by the relation ${\gamma }_{-CH2-}$= 52.603–0.058 T for all temperatures.

#### Critics of the classic methods^18^

It is obvious, in the two previous methods based on Fowkes relation, that the determination of the dispersive surface energy component ${\gamma }_{s}^{d}$ of a solid, necessitates the precise knowledge of the surface areas, *a,* of n-alkanes adsorbed on the solid substrate. However, the surface area of a molecule adsorbed on a solid is not known with a good accuracy due to the large dependency on the temperature change. In a previous study, Hamieh and Schultz^18^ criticized the classical way and proposed to use various models giving the molecular areas of n-alkanes. The geometrical model, cylindrical molecular model, liquid density model, BET method, Kiselev results and the model using the two-dimensional Van der Waals (VDW) constant *b* that depends on the critical temperature and pressure of the liquid were considered. Redlich–Kwong (R–K) equation transposed from three-dimensional space to two-dimensional space was also used to calculate the areas of organic molecules. The value of ${\gamma }_{s}^{d}$ depends significantly on the chosen molecular models of the surface area of n-alkanes and on the temperature. The different molecular models for the different n-alkanes are listed in Table 1.

**Table 1:** Surface areas of various molecules (in Å^2^) obtained from the various models of Van der Waals (VDW), Redlich–Kwong (R–K) and Kiselev models.

Molecule	VDW	Kiselev	Cylindrical	R-K	Spherical	Geometrical
C_5_H_12_	47.0	45	39.3	36.8	36.4	32.9
C_6_H_14_	52.7	51.5	45.5	41.3	39.6	40.7
C_7_H_16_	59.2	57	51.8	46.4	42.7	48.5
C_8_H_18_	64.9	63	58.1	50.8	45.7	56.2
C_9_H_20_	69.6	69	64.4	54.5	48.7	64.0
C_10_H_22_	74.4	75	70.7	58.2	51.7	71.8

The surface areas are also compared to those obtained by geometrical, cylindrical or spherical models.

**Figure 3:**
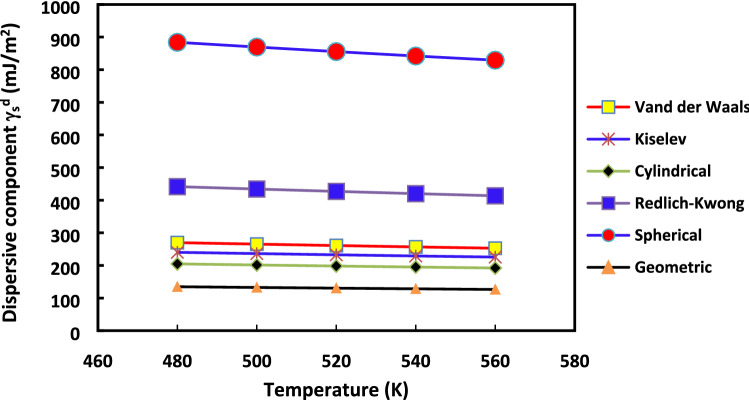
Evolution of ${\gamma }_{s}^{d}$(*T*) versus the temperature for the various molecular models: Van der Waals, Redlich–Kwong, Kiselev, geometrical, cylindrical and spherical models, by using the increment method for H-β-zeolite.

**Figure 4:**
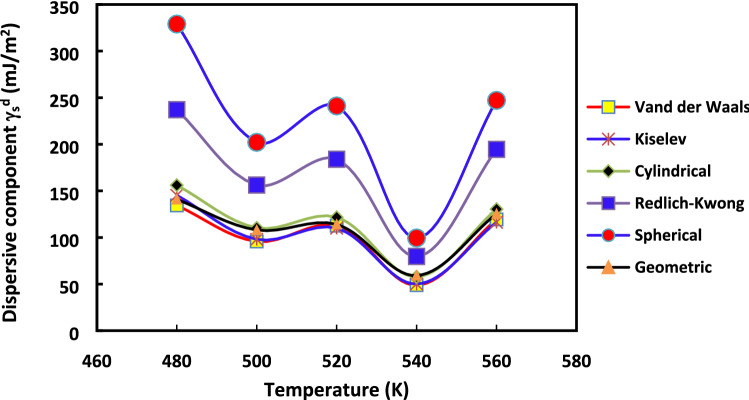
Evolution of ${\gamma }_{s}^{d}$(*T*) versus the temperature for the various molecular models: Van der Waals, Redlich–Kwong, Kiselev, geometrical, cylindrical and spherical models, by using the straight-line method for H-β-zeolite.

It appears relevant to strengthen our analysis and to show the effect of the method used and the molecular models chosen on ${\gamma }_{s}^{d}$ values. The variations of ${\gamma }_{s}^{d}$ as a function of the temperature for the various molecular models of n-alkane surface areas are displayed, respectively, in the case of the increment method (Fig. 3) and the straight methods (Fig. 4).

Figures 3 and 4 clearly highlighted the extreme variation of ${\gamma }_{s}^{d}$(*T*) depending on the chosen method and molecular model of the surface area of n-alkanes. The difference between the values of ${\gamma }_{s}^{d}$ reached about 100% from Kiselev, van der Waals or cylindrical models to Redlich–Kwong or spherical models for all used temperatures regardless of the method (increment method and straight-line method). However, for any method and molecular model used, some physico-chemical behavior can be deduced when comparing the surface energy of two solid materials. This can be useful to understand the change of catalyst properties when the temperature varies.

### Determination of the specific interactions

The free energy of adsorption *∆G*^*0*^ of a probe on a solid generally contains the two contributions relative to the dispersive and specific interactions. In the case of n-alkanes, *∆G*^*0*^ is equal to the free energy of adsorption corresponding to the dispersive interactions *∆G*^*d*^ only. When polar molecules are injected into the column, specific interactions are established between these probes and the solid surface and *∆G*^*0*^ is now given by:

13 $$ \Delta G^{0} = \Delta G^{d} + \Delta G^{sp} . $$

where *∆G*^*sp*^ refers to specific interactions of a polar molecule adsorbed on solid substrate.

To calculate the specific interactions between the solid substrates and polar probes, several methods were used in the literature^5–8,18,19,27–29^. To avoid the use of the method based on the surface area of n-alkanes that cannot be known precisely as a function of the temperature, the method developed by Papier et al.^29^ is preferred. It allows to quantify more precisely the specific interactions.

#### Saint Flour and Papirer's method

Papirer method is employed to quantify the specific free energy of adsorption of polar molecules and obtain the acid–base constants of the different hydrocarbon materials. This method gives access to the specific enthalpy of interaction between a probe and a solid^27–29^ from the obtained straight line when plotting *RTlnVn* against the logarithm of the vapor pressure of the probes*, i.e. lnP*_*0*_.

For a homologous series of n-alkanes, whatever the nature of the solid substrates:

14 $$ RT \, lnVn \, \left( {n - alkane} \right) \, = \, A \, lnP_{0} \left( {n - alkane} \right) \, + \, B $$

where *A* and *B* are constants which depend on the nature of the solid substrate.

**Figure 5:**
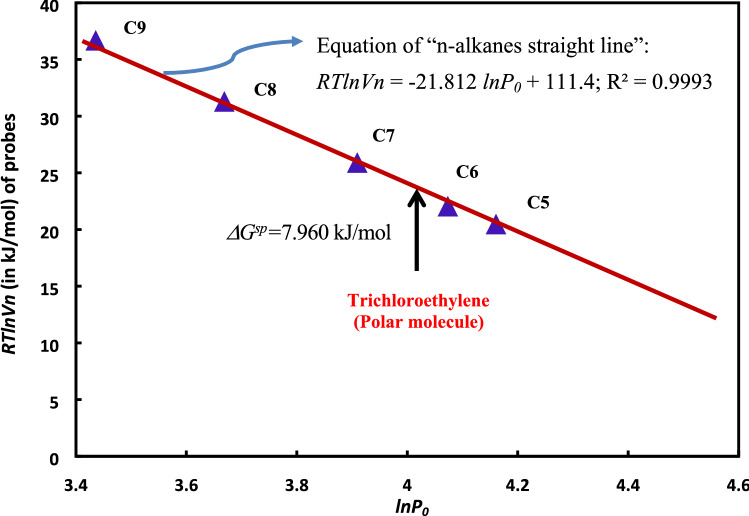
Variations of *RTlnVn* of n-alkanes and polar probes as a function of *lnP*_*0*_ in the case of H-β-zeolite at 480 K.

Following Saint Flour and Papirer's approach^26,27^, *RTlnVn* values of the various solutes are first plotted versus the logarithm of their vapor pressure at saturation, *Po*. The points representative of n-alkanes define the so-called “n-alkane straight line” (see Fig. 5), and the distance between this line and the points corresponding to *RTlnVn* (*polar molecule*) value of polar probes are then taken as a measure of the specific interactions and it is defined as the specific free enthalpy of adsorption, *∆G*^*s*^*,* of polar molecule on the solid. It is given, for any temperature *T*, by the following equation:

15 $$ \Delta G^{sp} \left( {polar \, molecule} \right) = RTlnVn\left( {polar \, molecule} \right) \, - A \, lnP_{0} \left( {polar \, molecule} \right) - \, B $$

As example, the variations of *RTlnVn* of different n-alkanes and polar probes as a function of *lnP*_*0*_ in the case of H-β-zeolite at 480 K are reproduced in the Fig. 5. The equation of n-alkanes straight line is given with an excellent linearity:

16 $$ RT\ln Vn = - 21.812\ln P_{0} + {\mkern 1mu} 111.4;{\text{ R}}{\mkern 1mu} = {\mkern 1mu} 0.9993 $$

From this equation, the specific free enthalpy of adsorption of polar molecules can be deduced. For example, for trichloroethylene *∆G*^*sp*^ is equal to 7.960 kJ/mol.

In the following, the specific free enthalpy of adsorption of polar probes *∆G*^*s*^*(T)* can be determined by varying the temperature. The corresponding values of *(∆H*^*sp*^*)* and entropy *∆S*^*sp*^ of adsorption of polar molecules are obtained.

### Determination of acid–base constants of solid substrates

By plotting *∆G*^*sp*^*(T)* of the polar molecules as a function of the temperature, the specific enthalpy.

*(∆H*^*sp*^*)* and entropy *∆S*^*sp*^ of adsorption are calculated from:

17 $$ \Delta G^{s} \left( T \right) = \Delta H^{sp} - \, T\Delta S^{sp} $$

**Figure 6:**
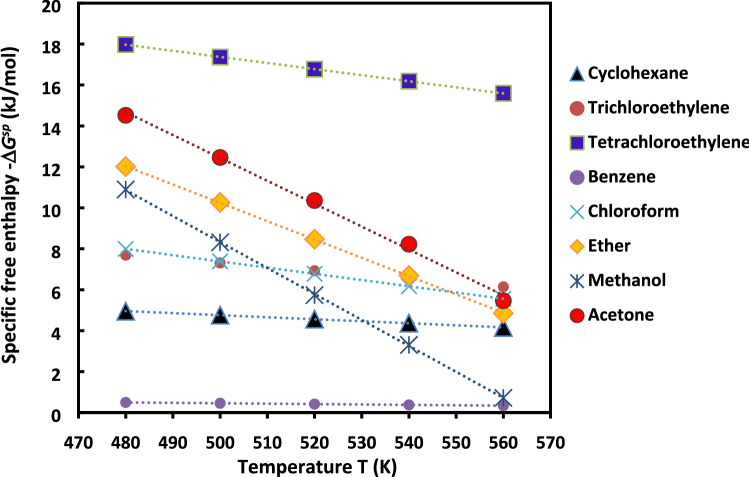
Curves of -*∆G*^*sp*^*(T)* of polar molecules as a function of the temperature in the case of H-β-zeolite for a range temperature [480 K, 560 K].

The evolution of *∆G*^*sp*^*(T)* of the polar molecules as a function of the temperature in the case of H-β-zeolite is plotted in Fig. 6. In general, this relationship (17) is linear if *∆H*^*sp*^ and *∆S*^*sp*^ do not depend on the temperature. However, when the linear correlation coefficient is too small in front of 1, then the linearity is not verified; therefore, *∆H*^*sp*^*(T)* and *∆S*^*sp*^*(T)* strongly depend on the temperature. The curves representing the variations of *∆G*^*sp*^*(T)* versus the temperature give access to the thermodynamic calculations of specific enthalpy and entropy as a function of the temperature by using the classical thermodynamic equations. The specific enthalpy and entropy of adsorption determined from the linear relation between *∆G*^*sp*^ and T are summarized in the Table 2.

**Table 2:** Values of the specific enthalpy − *∆H*^*sp*^ and *∆S*^*sp*^ entropy of adsorption of polar molecules adsorbed on H-β-zeolite substrate.

Polar probes	Specific enthalpy − *∆H*^*sp*^ (kJ/mol)	Specific entropy *∆S*^*sp*^ (J/mol)
Cyclohexane	9.713	− 10
Trichloroethylene	16.960	− 19
Tetrachloroethylene	32.242	− 30
Benzene	1.457	− 2
Chloroform	22.592	− 30
Ether	54.982	− 90
Methanol	71.74	− 127
Acetone	68.321	− 112

#### The Guttmann method

Gutmann^33^ classified the polar molecules by assigning an electron donor (*DN*) and a number of electron acceptor (*AN*) which realize, respectively, the acidity and the basicity of the molecule. In analogy to the Guttmann approach, Papirer et al.^27–29^ proposed to characterize the solid by two parameters. The parameters *K*_*A*_ and *K*_*D*_ reflect the basic and the acidic character of the solid, respectively. These two constants measure the ability of the solid to develop, respectively, the acid and base interactions with basic, acidic or amphoteric probes. They are connected to the specific enthalpy Δ*H*_*a*_^*SP*^ through the following equation:

18 $$ ( - DH^{sp} ) \, = \, \left( {K_{A} .DN \, + \, K_{D} .AN} \right) $$

where *K*_*A*_ and *K*_*D*_ represent the acidic and the basic character of the solid, respectively, while *AN* and *DN* represent the donor number and the electron acceptor of the probe according to the scale of Gutmann^33^.

Equation 11 can be rewritten as:

19 $$ \frac{{ - \Delta H^{Sp} }}{AN} = \frac{DN}{{AN}} K_{A} + \, K_{D} $$

The representation of $\frac{-\Delta {H}^{Sp}}{AN}$ as a function of $\frac{DN}{AN}$ gives, in general, a straight line of slope *K*_*A*_ and intercept *K*_*D*_.

#### The new model

For several solid substrates, the Guttmann method cannot be applied because the linearity of Eq. 19 is not satisfied. This classical relationship was corrected. Then, a new equation was proposed^18,19^. A third parameter *K* was added. It reflected the amphoteric character of the oxide or polymer. The final expression becomes:

20 $$ ( - \Delta Hsp) \, = K_{A} DN \, + \, K_{D} AN{-}K \, DN \, AN $$

By dividing by *AN*, one can obtain:

21 $$ { - }\frac{{\Delta H^{sp} }}{AN} = K_{A} \frac{DN}{{AN}} + K_{D} { - }K_{{}} {.}D{\text{N}} $$

The Eq. (21) can be symbolically written as:

22 $$ X{1} = K_{D} + K_{A} X{2} - K \, X{3} $$

where $$X_{1} = { - }\frac{{\Delta H^{sp} }}{AN}$$,$$X_{2} = \frac{DN}{{AN}}$$, $$X_{3} = DN$$ and *K* = *K*(*K*_*A*_*,K*_*D*_).

Note that *X*_1_, *X*_2_ and *X*_3_ are known for every polar molecule, whereas *K*_*D*_, *K*_*A*_ and *K* are unknown. By using *N* probes, relationship (22) leads to a set of linear system of three equations with three unknown variables: *K*_*D*_, *K*_*A*_ and *K*. The matrix representing this linear application is a symmetrical one. It appears that Eq. (22) possesses a unique solution for *N* ≥ 3. This method can be applied to calculate the acid–base constants of solids if the Gutmann relation falls.

## Experimental results on rhodium supported by H-β-zeolite

### Materials and solvents

The different catalysts analyzed in this study containing rhodium supported by H-β-zeolite were obtained by following the method developed by Navio et al.^24^ and Zhang et al.^25^ to have different percentages of rhodium. Classical organic probes, characterized by their donor and acceptor numbers, were used. Corrected acceptor number *AN′* = *AN–AN*^*d*^ were utilized. They were given by Riddle and Fowkes^34^. The idea was to subtract the contribution of Van der Waals interactions (or dispersion forces). The corrected acceptor number was then normalized by a dimensionless donor number *DN′* according to the following relationship^18,19^:

23 $$ DN\prime = { 2}.{5}\left( {{\text{mol}}/{\text{kcal}}} \right)DN\left( {{\text{kcal}}/{\text{mol}}} \right) $$

However, if one wants to use *DN* in kcal/mol, *AN′* can be easily transformed to the kcal/mol unit using the following relationship:

24 $$A{N}^{^{\prime}}(kcal/mol)= \frac{40 \left(kcal/mol\right)}{100} A{N}^{^{\prime}} (\mathrm{unitless})$$

The solvents used as probes for IGC measurements were selected based on their ability to interact with three different types of interaction forces, namely dispersive, polar, and hydrogen bonding. All probes were obtained from Aldrich. They were highly pure grade (i.e., 99%). The probes used were n-alkanes (pentane, hexane, heptane, octane, and nonane), amphoteric solvents (methanol, acetone, trichloroethylene (Tri-CE), tetrachloroethylene (Tetra-CE)), strong basic solvent (diethyl ether), very weak basic solvent (benzene), very acidic solvent (chloroform), and very weak acid (cyclohexane). The Table 3 gives the donor and acceptor numbers^18,33^ of polar probes used in this study.

**Table 3:** Normalized donor and acceptor numbers of some polar molecules.

Polar probe	DN'	AN'	DN'/AN'	Character
Chloroform	18.600	0.000	0.000	Higher acidity
Ether	4.900	48.000	9.796	Higher basicity
Methanol	41.700	47.500	1.139	High amphoteric
Acetone	8.700	42.500	4.885	High amphoteric
Cyclohexane	0.141	3.520	24.965	Weaker acidity
Trichloroethylene	3.313	2.500	0.755	Weak amphoteric
Tetrachloroethylene	3.020	3.100	1.026	Weak amphoteric
Benzene	0.600	0.250	0.417	Weak acidity and basicity

### GC conditions

The IGC measurements were performed on a commercial Focus GC gas chromatograph equipped with a flame ionization detector. Dried nitrogen was the carrier gas. The gas flow rate was set at 20 mL/min. The injector and detector temperatures were maintained at 400 K during the experiments^30^. To achieve infinite dilution, 0.1 μL of each probe vapor was injected with 1 μL Hamilton syringes, in order to approach linear condition gas chromatography. All four columns used in this study were prepared using a stainless-steel column with a 2 mm inner diameter and with an approximate length of 20 cm. The column was packed with 1 g of solids in powder forms. In general, the surface properties of materials are studied by IGC at low temperatures. However, in certain case for lower temperatures, the retention times of organic molecules are very long due to the difficulties for the probes to find its path through the catalyst particles. For this reason, the experiments were conducted at higher temperatures in order to quantify the surface properties of catalysts by IGC at infinite dilution and deduce the acid base and dispersive surface energy of solid substrates. The column temperatures were 480–560 K, varied in 20 °C steps. Each probe injection was repeated three times, and the average retention time, *t*_*R*_, was used for the calculation. The standard deviation was less than 1% in all measurements.

### Results and discussion

#### Variations of the net retention volume

Experimental results obtained by IGC at infinite dilution with different percentages of rhodium (from 0 to 2%) supported by H-β-zeolite at various temperatures (from 480 to 560 K), are presented in Tables 4, 5, 6, 7, 8.

**Table 4:** Values of *RTlnVn* (in kJ/mol) of n-alkanes and polar probes for different percentages of rhodium supported by H-β-zeolite substrate (%Rh = 0; 0.25; 0.50; 0.75; 1.00; 1.25; 1.50; 1.75; 2.00) at T = 480 K.

%Rh/H-β-Z probes	0	0.25	0.5	0.75	1	1.25	1.5	1.75	2
C5	15.117	16.78	17.841	18.1	17.879	17.1	16.41	16.2	16.172
C6	20.481	22	23.35	24	23.342	22.2	21.7	21.6	21.523
C7	25.909	27.62	28.836	29.453	28.62	27.356	26.8	26.65	26.624
C8	31.294	32.854	34.337	35.116	34.021	32.511	32.012	31.912	31.892
C9	36.69	38.523	39.835	40.21	39.392	38.111	37.355	37.154	37.118
Cyclohexane	18.243	19.421	20.528	21.335	20.831	19.731	18.995	18.864	18.834
Tri-CE	16.082	18.025	19.560	22.613	24.406	21.311	18.461	18.370	18.331
Tetra-CE	18.046	20.147	24.566	31.244	33.136	29.277	20.608	19.997	19.947
Benzene	22.054	23.421	24.618	25.447	25.207	22.305	22.301	22.454	22.536
Chloroform	11.257	10.684	10.367	11.500	10.548	9.766	9.336	9.217	9.166
Ether	1.35	5.750	6.550	7.860	8.750	8.120	7.810	7.230	7.150
Methanol	2.000	7.240	8.450	9.850	10.560	9.870	8.450	8.012	7.980
Acetone	1.876	9.230	9.780	10.230	10.840	10.450	9.380	9.045	8.986

**Table 5:** Values of *RTlnVn* (in kJ/mol) of n-alkanes and polar probes for different percentages of rhodium supported by H-β-zeolite substrate (%Rh = 0; 0.25; 0.50; 0.75; 1.00; 1.25; 1.50; 1.75; 2.00) at T = 500 K.

%Rh/H-β-Z probes	0	0.25	0.5	0.75	1	1.25	1.5	1.75	2
C5	13.701	15.275	16.619	17.254	16.541	15.740	15.102	14.900	14.814
C6	18.879	20.500	21.880	22.500	21.840	20.740	20.310	20.210	20.137
C7	24.145	25.777	27.192	28.078	26.956	25.612	25.178	25.100	25.006
C8	29.352	31.021	32.470	33.153	32.194	30.979	30.300	30.211	30.178
C9	34.574	36.200	37.757	38.362	37.402	36.000	35.530	35.370	35.274
Cyclohexane	4.169	16.821	18.000	19.070	19.688	19.327	18.711	18.182	17.930
Tri-CE	4.149	14.704	16.700	18.406	21.828	23.249	20.309	17.477	17.150
Tetra-CE	3.612	16.688	18.632	23.203	30.123	31.907	28.241	19.348	18.800
Benzene	4.200	20.419	21.761	23.104	24.123	23.645	20.740	20.830	21.024
Chloroform	4.343	10.293	9.945	9.755	10.612	10.291	9.522	8.780	8.601
Ether	4.603	1.570	6.250	6.780	7.230	7.789	7.641	7.230	7.100
Methanol	4.664	2.150	8.260	8.887	10.220	10.856	9.680	9.350	9.159
Acetone	4.480	2.150	8.740	9.210	10.230	10.840	10.450	10.184	9.879

**Table 6:** Values of *RTlnVn* (in kJ/mol) of n-alkanes and polar probes for different percentages of rhodium supported by H-β-zeolite substrate (%Rh = 0; 0.25; 0.50; 0.75; 1.00; 1.25; 1.50; 1.75; 2.00) at T = 520 K.

%Rh/H-β-Z probes	0	0.25		0.5		0.75		1		1.25		1.5		1.75		2	
C5	12.285	14.022		15.397		15.897		15.203		14.300		13.598		13.511		13.456	
C6	17.277	19.000		20.410		21.023		20.338		19.526		19.000		18.878		18.751	
C7	22.381	23.989		25.548		26.101		25.292		24.200		23.587		23.460		23.388	
C8	27.410	29.230		30.603		31.489		30.367		29.454		28.845		28.566		28.464	
C9	32.458	34.213		35.679		36.340		35.411		34.360		33.689		33.520		33.430	
Cyclohexane	15.399	16.630		17.612		18.154		17.823		17.420		17.100		16.922		16.914	
Tri-CE	13.326	15.378		17.252		20.797		22.094		19.469		16.120		15.900		15.819	
Tetra-CE	15.330	17.200		21.843		28.994		30.697		27.588		18.237		17.612		17.463	
Benzene	18.788	20.229		21.597		22.537		22.090		19.334		19.352		19.566		19.622	
Chloroform	9.328	9.130		9.144		9.500		10.034		9.000		8.142		8.000		7.910	
Ether	1.850	2.650		3.221		3.678		4.623		4.232		4.012		3.877		3.798	
Methanol	2.350	3.456		4.412		4.941		5.714		5.245		5.014		4.886		7.778	
Acetone	2.480	4.124		4.876		5.664		6.356		5.884		5.665		5.514		5.412

**Table 7:** Values of *RTlnVn* (in kJ/mol) of n-alkanes and polar probes for different percentages of rhodium supported by H-β-zeolite substrate (%Rh = 0; 0.25; 0.50; 0.75; 1.00; 1.25; 1.50; 1.75; 2.00) at T = 540 K.

%Rh/H-β-Z probes	0	0.25		0.5		0.75		1		1.25		1.5		1.75		2	
C5	10.869	12.700		14.175		15.211		13.865		12.890		12.350		12.200		12.098	
C6	15.675	17.456		18.940		19.356		18.836		18.211		17.660		17.456		17.365	
C7	20.617	22.411		23.904		24.789		23.628		22.678		22.100		21.900		21.770	
C8	25.468	27.190		28.735		29.455		28.539		27.500		26.997		26.860		26.750	
C9	30.342	32.233		33.600		34.300		33.421		32.300		31.800		31.677		31.586	
Cyclohexane	13.977	15.200		16.154		16.520		16.319		16.080		16.050		16.000		15.954	
Tri-CE	11.948	14.120		16.098		19.993		20.930		18.461		14.950		14.697		14.563	
Tetra-CE	13.972	15.900		20.495		27.727		29.490		26.493		16.934		16.333		16.221	
Benzene	17.154	18.665		20.081		21.212		20.530		17.737		17.923		18.120		18.166	
Chloroform	8.364	8.400		8.532		9.078		9.778		8.700		7.543		7.355		7.282	
Ether	2.115	2.733		3.420		3.876		3.456		3.117		2.977		2.778		2.612	
Methanol	2.450	3.012		3.656		4.031		3.678		3.312		3.185		3.033		2.897	
Acetone	2.851	3.654		4.221		4.785		4.335		3.915		3.687		3.421		3.334

**Table 8:** Values of *RTlnVn* (in kJ/mol) of n-alkanes and polar probes for different percentages of rhodium supported by H-β-zeolite substrate (%Rh = 0; 0.25; 0.50; 0.75; 1.00; 1.25; 1.50; 1.75; 2.00) at T = 560 K.

%Rh/H-β-Z probes	0	0.25	0.5	0.75	1	1.25	1.5	1.75	2
C5	9.453	11.500	12.953	13.220	12.527	11.500	10.950	10.800	10.740
C6	14.073	15.870	17.470	18.000	17.334	16.600	16.166	16.070	15.979
C7	18.853	20.700	22.260	22.700	21.964	21.100	20.396	20.266	20.152
C8	23.526	25.417	26.868	27.700	26.712	25.800	25.290	25.159	25.036
C9	28.226	30.000	31.522	32.344	31.431	30.500	29.961	29.822	29.742
Cyclohexane	12.555	13.770	14.696	15.200	14.815	14.850	14.930	14.962	14.994
Tri-CE	10.570	12.900	14.944	18.779	19.770	17.450	13.700	13.412	13.307
Tetra-CE	12.614	14.581	19.149	26.725	28.295	25.647	15.700	15.025	14.979
Benzene	15.513	17.128	18.568	19.425	18.975	16.238	16.378	16.636	16.707
Chloroform	7.399	7.566	7.920	8.580	9.521	8.350	6.970	6.712	6.654
Ether	2.452	3.687	4.256	4.904	5.371	5.046	4.786	4.660	4.589
Methanol	2.750	3.425	3.879	4.623	4.970	4.572	4.364	4.271	4.182
Acetone	3.851	4.456	5.012	5.706	6.041	5.731	5.493	5.322	5.278

**Figure 7:**
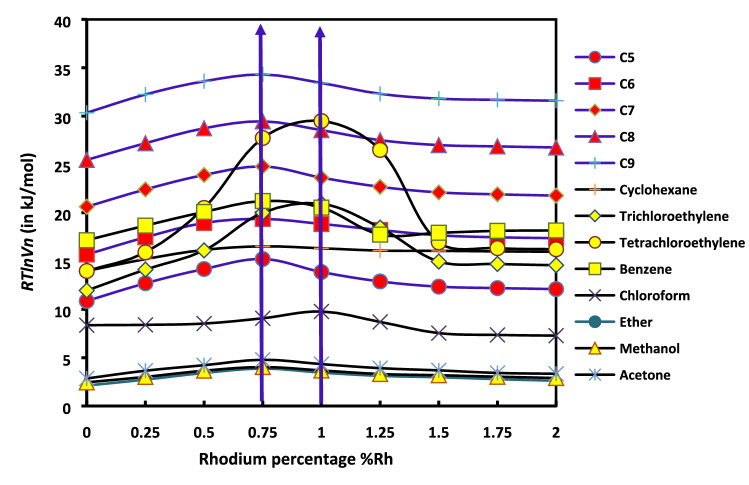
Variations of *RTlnVn* of n-alkanes and polar probes adsorbed on the solid substrates as a function of the percentage of rhodium supported by H-β-zeolite (%Rh).

The Tables indicate substantial variations of *RTlnVn* between the probes adsorbed on the solid substrates. Consequently, significant variations of the surface free enthalpy of adsorption are expected. This aspect is emphasized in the Fig. 7.

It is interesting to note a particular point represented by a maximum of *RTlnVn*. In the case of n-alkanes adsorbed on the catalysts it takes place for a percentage of rhodium %Rh/H-β-Z = 0.75% (Fig. 7). However, this maximum of the surface free enthalpy shifts to a percentage %Rh/H-β-Z of 1.00% in the case of polar solvents. This shift maybe attributed to the strong specific interaction of the polar molecules with rhodium.

**Figure 8:**
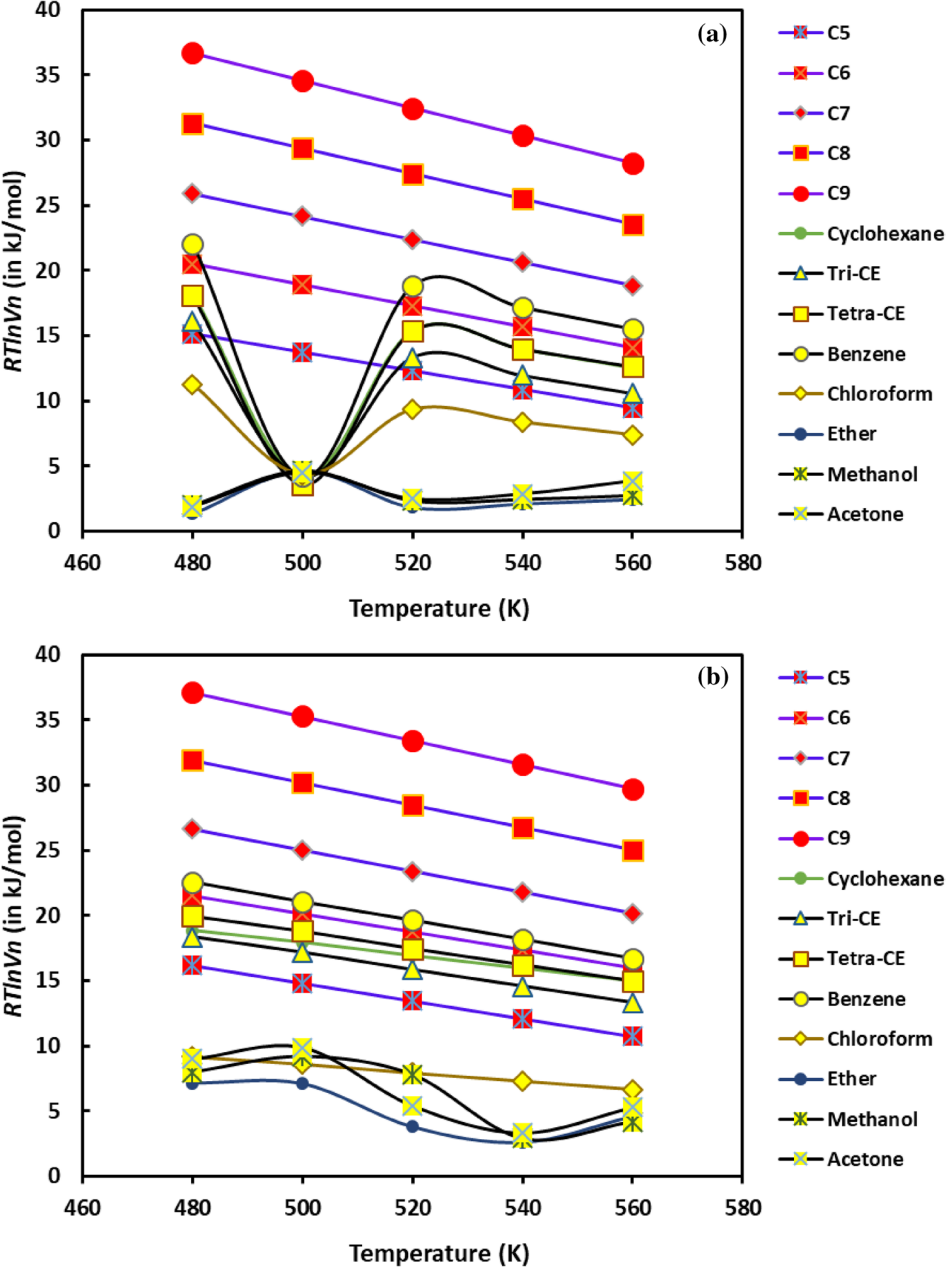
Variations of *RTlnVn* of n-alkanes and polar probes adsorbed on the solid substrates as a function of the temperature *T* (*K*) in the case of (**a**) H-β-zeolite and (**b**) rhodium supported by H-β-zeolite (2%Rh).

The evolution of *RTlnVn* as a function of the temperature for n-alkanes and polar molecules is given in the Fig. 8. In the case of H-β-zeolite substrate, a linear dependency for all alkane solvents is observed (Fig. 8a). Conversely, for all the polar molecules, a non-linear behavior occurs with a minimum for T = 500 K where the surface groups of the solid substrate are strongly affected by the temperature change. The same behavior takes place with all the polar molecules on H-β-zeolite at 500 K. At this temperature, they have identical resident or retention time due to a minimum polarity of the catalyst at this temperature leading to weak polar interactions between the probes and the H-β-zeolite.

The dispersive interactions can be considered similar for all polar molecules in this case. This gives similar values of *RTlnVn* at T = 500 K. It seems that, at this temperature, some surface groups of H-β-zeolite are inaccessible for polar probes that cross more quickly the chromatographic column. In addition, when the temperature increases, the acid base surface groups of the solid increases. Consequently, the values of *RTlnVn* also increase for all the polar molecules.

In the presence of rhodium incorporated into H-β-zeolite catalyst, the minimum of *RTlnVn* with the temperature disappears for polar molecules (Fig. 8b). In addition, a global linear tendency is observed for polar and non-polar molecules. The presence of the rhodium particles on the surface of H-β-zeolite catalyst affects strongly the specific interactions between the polar molecules and the catalyst while whereas the dispersive interactions remain stable and constant.

However, in order to better quantify the specific free enthalpy of interaction between the catalyst and the polar molecules, the classical thermodynamic equations are used in the “Determination of the specific interactions and acid–base properties”. The obtained specific free enthalpy of adsorption gives a real idea of the nature of acid base interactions at any temperature.

#### Determination of the dispersive component of the surface energy of catalysts

In this section, the dispersive component of the surface energy of the rhodium supported by H-β-zeolite are determined at different percentages of Rh and for various temperatures. The various surface areas of n-alkanes given by the different molecular models are also used knowing the values of the dispersive component of the surface energy ${\gamma }_{l}^{d} (T)$ of n-alkanes as a function of the temperature. The calculations of ${\gamma }_{s}^{d}$(*T*) are performed using the increment method of Dorris and Gray and the method of the straight-line for all solid substrates. They are calculated at different temperatures and molecular models. The results obtained by using Dorris and Gray method are listed on Table 9 while those calculated thanks to the straight-line method are given in Table 10. They are estimated for different temperatures, rhodium percentages and molecular models.

**Table 9:** Values of ${\gamma }_{s}^{d}$ of different catalysts as a function of temperature, rhodium percentage and molecular model using the increment method.

**%Rh**	**T (K)**	**Van der Waals**		**Kiselev**		**Cylindrical**		**Redlih–Kwong**		**Spherical**		**Geometric**	
0% (H-β-zeolite)	480	270		240		205		442		*884*		135	
	500	265		236		201		434		*870*		133	
	520	261		233		198		427		*856*		130	
	540	257		229		195		420		*842*		128	
	560	253		226		192		414		*829*		126	
0.25%	480	283		250		213		462		919		140	
	500	265		237		202		433		870		133	
	520	262		233		199		429		861		131	
	540	263		233		198		430		855		130	
	560	250		225		192		409		826		126	
0.50%	480	280		248		212		457		915		139	
	500	272		242		206		445		890		136	
	520	264		235		201		432		865		132	
	540	256		229		195		419		841		128	
	560	249		223		189		407		817		125	
0.75%	480	266		241		205		436		886		135	
	500	269		243		206		439		888		136	
	520	263		237		202		431		873		133	
	540	263		241		203		429		875		134	
	560	259		231		198		425		854		130	
1.00%	480	266		235		201		435		868		132	
	500	262		232		198		429		856		130	
	520	259		229		195		423		844		129	
	540	255		226		193		418		834		127	
	560	252		223		191		413		824		126	
1.25%	480	266		231		198		435		854		130	
	500	252		223		191		413		825		126	
	520	252		221		190		412		821		125	
	540	241		210		180		394		780		119	
	560	247		217		185		404		802		122	
1.50%	480	255		223		191		417		826		126	
	500	254		221		190		415		820		125	
	520	247		217		186		405		806		123	
	540	243		212		182		398		786		120	
	560	245		213		183		402		794		121	
1.75%	480	251		221		189		410		815		124	
	500	251		220		188		410		813		124	
	520	247		215		185		404		800		122	
	540	246		214		184		402		796		121	
	560	244		211		182		400		789		120	
2.00%	480	251		222		190		411		819		125	
	500	251		220		188		410		813		124	
	520	248		216		186		406		803		122	
	540	246		214		184		403		796		121	
	560	246		212		183		402		791		120

**Table 10:** Values of ${\gamma }_{s}^{d}$ of different catalysts as a function of temperature, rhodium percentage and molecular model using the straight-line method.

**%Rh**	**T (K)**	**Van der Waals**	**Kiselev**	**Cylindrical**	**Redlih–Kwong**	**Spherical**	**Geometric**
0%	480	134	145	156	237	329	141
	500	96	98	110	156	202	108
	520	113	110	122	184	241	114
	540	49	50	58	80	99	59
	560	119	116	130	195	247	125
0.25%	480	135	150	161	244	338	145
	500	96	98	111	157	203	108
	520	116	113	125	190	248	117
	540	50	51	59	81	101	60
	560	114	111	124	185	235	142
0.50%	480	139	150	162	245	341	146
	500	98	100	113	160	207	111
	520	114	111	123	186	244	115
	540	49	50	58	79	99	59
	560	117	114	128	191	242	123
0.75%	480	142	124	158	240	297	142
	500	97	100	112	159	206	110
	520	117	114	126	190	249	118
	540	47	48	55	79	95	57
	560	117	114	127	190	241	123
1.00%	480	131	143	154	233	323	139
	500	94	97	109	154	199	106
	520	114	111	123	186	243	115
	540	50	51	59	79	101	60
	560	120	117	131	196	249	126
1.25%	480	122	139	150	227	315	135
	500	91	92	106	149	193	103
	520	115	112	124	187	245	116
	540	48	49	57	78	97	58
	560	119	116	130	195	247	125
1.50%	480	122	136	146	221	307	132
	500	90	92	104	147	190	102
	520	113	111	122	185	243	115
	540	49	50	58	79	99	59
	560	118	115	129	192	244	124
1.75%	480	122	134	144	219	304	130
	500	89	91	103	146	188	101
	520	112	110	121	183	240	114
	540	50	51	59	81	101	60
	560	118	115	128	191	243	123
2.00%	480	135	135	145	220	306	131
	500	89	91	103	146	188	101
	520	112	109	121	183	240	113
	540	50	51	59	81	102	61
	560	120	117	131	195	248	126

Significant difference between the values of ${\gamma }_{s}^{d}$ obtained by the two applied methods can be noticed. This large difference in the values of ${\gamma }_{s}^{d}$ is due to the high temperatures neighboring 500 K. The surface tension of n-alkanes at such temperatures does not give an identical surface tension of the methylene group than that given by the classical relation (${\gamma }_{-CH2-}$= 52.603–0.058 T). The results also prove that ${\gamma }_{s}^{d}$ strongly depends on the molecular model chosen to estimate the surface areas of n-alkanes. Equation (11) can be also written as:

$${{\varvec{\gamma}}}_{{\varvec{s}}}^{{\varvec{d}}}= \frac{{\left(RTln{V}_{n}+C\right)}^{2}}{4{\mathcal{N}}^{2}{\gamma }_{l}^{d} {{\varvec{a}}}^{2}}$$

This equation clearly shows an important variation of ${\gamma }_{s}^{d}$ of a solid substrate as a function of the surface area *a* of molecules. Table 1 gave the different molecular models for the different n-alkanes and showed a larger variation of the surface area of molecules depending on the chosen molecular model. The standard deviation can rich in many cases more than 50% from a molecular model to another model. This leads to larger difference between the obtained values of ${\gamma }_{s}^{d}$ of a solid substrate at fixed temperature for the various molecular models. The value of ${\gamma }_{s}^{d}$ can vary from the simple to the double when passing from geometric model to the spherical model. This problem was solved by another study showing the variation of the surface areas of polar and n-alkane molecules as a function of the temperature^35^.

**Figure 9:**
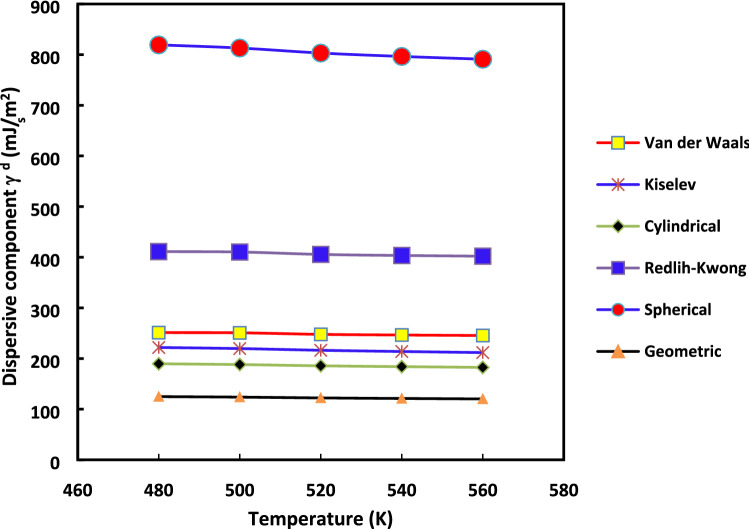
Evolution of ${\gamma }_{s}^{d}$(*T*) versus the temperature for the various molecular models: Van der Waals, Redlich–Kwong, Kiselev, geometrical, cylindrical and spherical models, by using the increment method in the case of 2%Rh catalyst.

**Figure 10:**
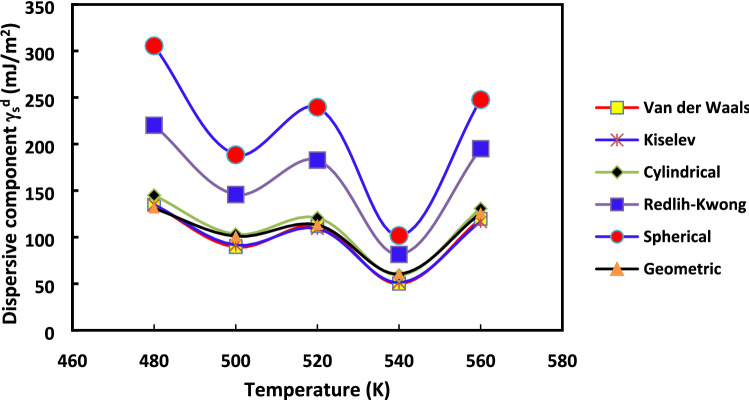
Evolution of ${\gamma }_{s}^{d}$(*T*) versus the temperature for the various molecular models: Van der Waals, Redlich–Kwong, Kiselev, geometrical, cylindrical and spherical models, by using the straight-line method in the case of 2%Rh catalyst.

The variations of ${\gamma }_{s}^{d}$ with the temperature in the case of H-β-Zeolite (for 0%Rh) were given previously in “Critics of the classic methods”. The aim here is to study the effect of the methods (increment or the straight-line methods) on the values of ${\gamma }_{s}^{d}$ with the temperature increases for the different molecular models in the case of 2%Rh catalyst. To this aim, the curves of ${\gamma }_{s}^{d}$(*T*) are plotted in in Fig. 9 for the case of where the increment method is used while the same curves with the straight-line method are given in Fig. 10. The same behavior is obtained with rhodium catalyst (supported by zeolite) as that of H-β-zeolite (without rhodium): linearity for the “increment method” and non-linearity for the “straight-line method”.

**Figure 11:**
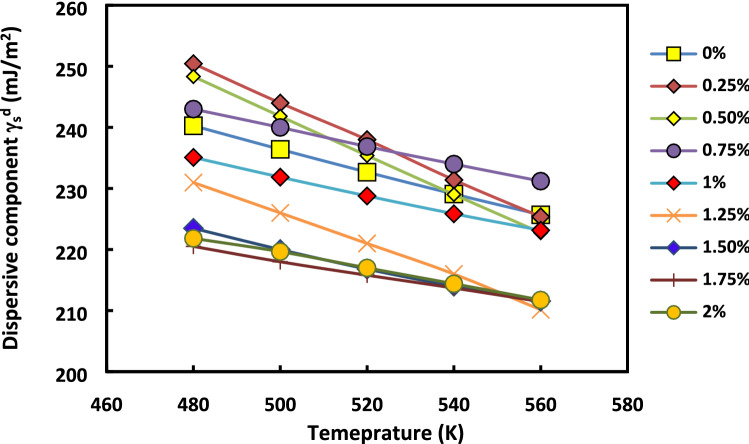
Evolution of ${\gamma }_{s}^{d}$(*T*) versus the temperature at various rhodium percentages by using the Dorris and Gray method and Kiselev molecular model.

**Figure 12:**
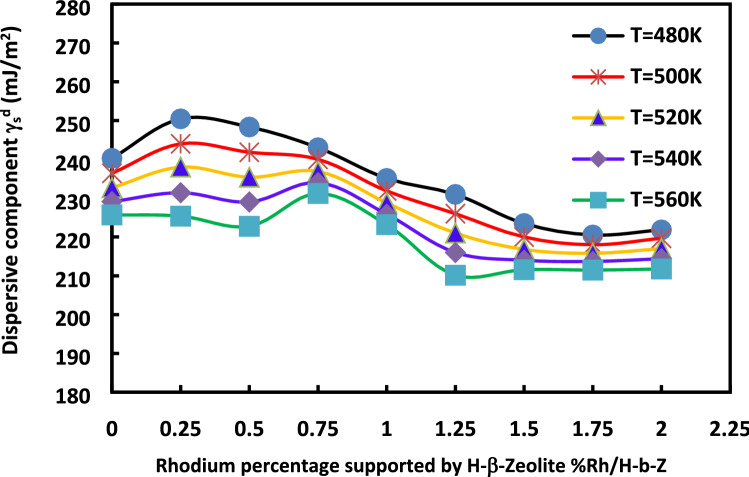
Evolution of ${\gamma }_{s}^{d}$(*T*) versus the rhodium percentage at various temperatures by using the Dorris and Gray method and Kiselev molecular model.

The rhodium percentage deposited on the H-β-zeolite has a high impact on the dispersive component of the surface energy of catalysts whatever the used temperature (Fig. 11) and molecular model (Fig. 12).

**Figure 13:**
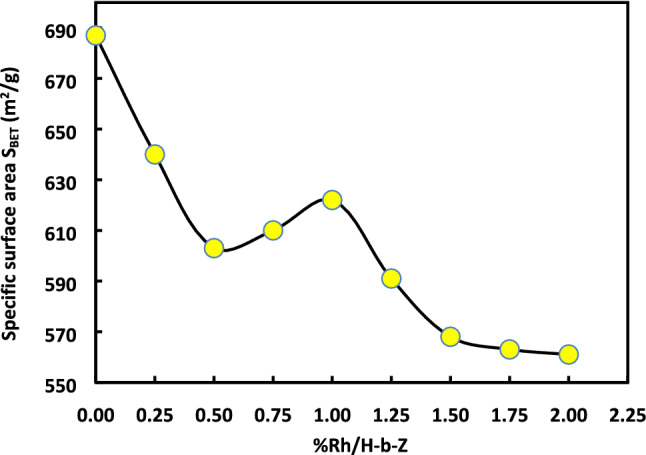
Variations of the specific surface area S_BET_ (m^2^/g) of catalysts versus the rhodium percentage %Rh.

It seems also relevant to evaluate the Variations of ${\upgamma }_{\mathrm{s}}^{\mathrm{d}}$ as a function of the specific surface area of the catalysts. Experimental results obtained by the BET method are presented in Table 11 and Fig. 13.

**Table 11:** Values of the specific surface area S_BET_ (m^2^/g) and microporous volume V_m_ (cm^3^/g) of the various catalyst samples.

%Rh	S_BET_ (m^2^/g)	V_m_ (cm^3^/g)
0	687	0.198
0.25	640	0.185
0.5	603	0.175
0.75	610	0.177
1	622	0.182
1.25	591	0.172
1.5	568	0.165
1.75	563	0.164
2	561	0.163

A non-linear decrease of the specific area with the amount of Rh occurs until 0.5%Rh. It is followed by a slight increase to reach a local maximum at 1%Rh. Then, the specific area decreases up to a plateau of specific surface area observed for %Rh larger than 1.50%. The same conclusion can be drawn for the microporous volume. However, the highest value of the specific surface area is obtained for H-β-zeolite.

**Figure 14:**
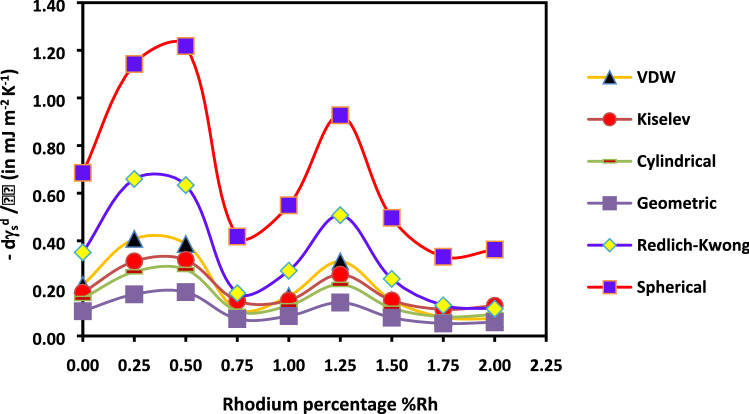
Variations of $-\frac{{d\gamma }_{s}^{d}}{dT}$ of catalysts versus the impregnated rhodium percentage (%Rh) for different molecular models of the surface area of n-alkanes.

It can be deduced from the figure that when the rhodium is added to zeolite, more metal particles would block the micropores causing a decrease in the specific surface area and in the catalyst microporosity. However, the increase of the specific surface area, for the catalysts containing a rhodium percentage comprised between 0.5 and 1.0, can result from the smaller particle sizes that cannot block the zeolite micropores. For catalysts with a rhodium percentage larger than 1.50% Rh, the lower surface area and pore volume are certainly due to the larger nanoparticles blocking the micropores and, then, decreasing the surface area and the pore volume. The curves of Fig. 14 giving the variations of—$\frac{{d\gamma }_{s}^{d}}{dT}$ as a function of the impregnated rhodium percentage give a similar behavior as that reported in the Fig. 13 for all the molecular models of n-alkane surface area.

### Determination of the specific interactions and acid–base properties

#### Variations of the specific free enthalpy

The experimental results obtained by IGC technique at infinite dilution previously presented in the Tables 4, 5, 6, 7, 8 lead to the determination of the specific free enthalpy *∆G*^*sp*^(*T*) of polar molecules adsorbed on H-β-zeolite and rhodium supported by zeolite for various temperatures and impregnated rhodium percentages. The results are summarized in Table 12.

**Table 12:** Values of the specific free enthalpy—*∆G*^*sp*^(*T*) (in kJ/mol) of different polar molecules adsorbed on catalysts as a function of the temperature and impregnated rhodium percentage.

**%Rh**	**T (K) polar probes**	**480**	**500**	**520**	**540**	**560**
0%	Cyclohexane	4.951	4.760	4.568	4.368	4.156
	Tri-CE	7.690	7.309	6.928	6.541	6.145
	Tetra-CE	17.975	17.366	16.772	16.183	15.592
	Benzene	0.497	0.457	0.417	0.377	0.337
	Chloroform	7.993	7.385	6.780	6.173	5.558
	Ether	12.012	10.264	8.468	6.694	4.850
	Methanol	10.896	8.317	5.738	3.307	0.720
	Acetone	14.520	12.460	10.357	8.222	5.463
0.25%	Cyclohexane	5.416	5.202	5.063	4.941	4.836
	Tri-CE	7.395	6.935	6.602	6.165	5.709
	Tetra-CE	17.606	17.080	16.658	16.064	15.407
	Benzene	0.769	0.734	0.700	0.662	0.625
	Chloroform	10.183	9.342	8.695	7.928	7.322
	Ether	11.327	9.679	7.985	6.313	4.574
	Methanol	9.262	7.070	4.877	2.811	0.612
	Acetone	11.616	9.968	8.286	6.578	4.370
0.50%	Cyclohexane	5.564	5.536	5.509	5.468	5.423
	Tri-CE	7.121	6.638	6.158	5.668	5.179
	Tetra-CE	14.595	14.030	13.480	12.915	12.351
	Benzene	0.819	0.789	0.759	0.729	0.699
	Chloroform	11.707	10.900	10.098	9.289	8.482
	Ether	10.682	9.127	7.530	5.953	4.313
	Methanol	7.872	6.009	4.146	2.389	0.520
	Acetone	9.293	7.975	6.629	5.262	3.496
0.75%	Cyclohexane	5.645	5.605	5.560	5.520	5.480
	Tri-CE	4.583	3.903	3.223	2.543	1.863
	Tetra-CE	8.521	7.801	7.081	6.361	5.641
	Benzene	0.496	0.456	0.416	0.376	0.336
	Chloroform	11.540	10.888	10.145	9.330	8.550
	Ether	10.073	8.607	7.101	5.614	4.067
	Methanol	6.692	5.108	3.524	2.031	0.442
	Acetone	7.434	6.380	5.303	4.210	2.797
1.00%	Cyclohexane	5.135	5.118	5.104	5.079	5.056
	Tri-CE	2.136	1.628	1.120	0.612	0.104
	Tetra-CE	5.597	4.977	4.357	3.737	3.117
	Benzene	0.119	0.097	0.075	0.053	0.031
	Chloroform	11.494	10.262	9.036	7.806	6.580
	Ether	9.499	8.116	6.696	5.294	3.835
	Methanol	5.688	4.342	2.995	1.726	0.376
	Acetone	5.948	5.104	4.242	3.368	2.238
1.25%	Cyclohexane	4.811	4.685	4.522	4.406	4.311
	Tri-CE	4.087	3.447	2.807	2.167	1.527
	Tetra-CE	7.996	7.216	6.436	5.656	4.876
	Benzene	1.909	1.910	1.902	1.945	1.870
	Chloroform	11.248	10.020	9.166	8.025	6.852
	Ether	8.957	7.654	6.315	4.992	3.617
	Methanol	4.835	3.690	2.546	1.467	0.319
	Acetone	4.758	4.083	3.394	2.694	1.790
1.50%	Cyclohexane	5.148	4.623	4.263	3.903	3.503
	Tri-CE	6.342	5.849	5.528	5.145	4.737
	Tetra-CE	16.044	15.600	15.156	14.712	14.268
	Benzene	1.320	1.288	1.256	1.224	1.192
	Chloroform	11.092	10.224	9.397	8.639	7.696
	Ether	8.447	7.217	5.955	4.707	3.410
	Methanol	4.109	3.137	2.164	1.247	0.272
	Acetone	3.806	3.266	2.715	2.155	1.432
1.75%	Cyclohexane	5.225	4.741	4.301	3.781	3.338
	Tri-CE	6.278	5.943	5.606	5.227	4.892
	Tetra-CE	16.506	16.039	15.562	15.169	14.807
	Benzene	1.012	0.959	0.906	0.853	0.801
	Chloroform	11.055	10.262	9.423	8.647	7.822
	Ether	7.965	6.806	5.615	4.439	3.216
	Methanol	3.493	2.666	1.840	1.060	0.231
	Acetone	3.045	2.613	2.172	1.724	1.146
2.00%	Cyclohexane	5.214	4.720	4.223	3.723	3.217
	Tri-CE	6.277	5.941	5.601	5.257	4.907
	Tetra-CE	16.525	16.062	15.614	15.177	14.745
	Benzene	0.889	0.827	0.765	0.703	0.641
	Chloroform	11.063	10.247	9.431	8.616	7.797
	Ether	7.511	6.418	5.295	4.186	3.033
	Methanol	2.969	2.266	1.564	0.901	0.196
	Acetone	2.436	2.090	1.738	1.379	0.917

**Figure 15:**
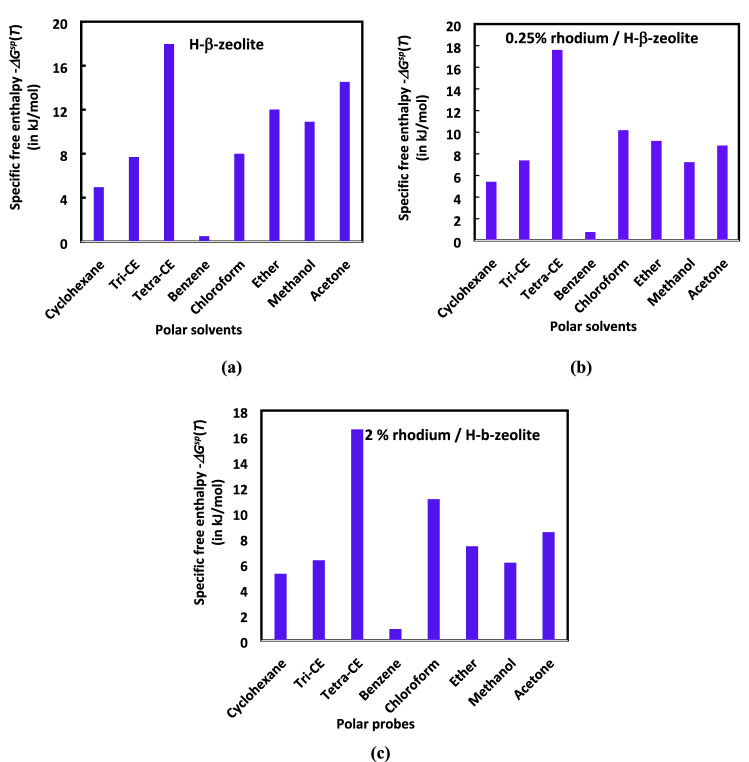
Comparison between the specific free enthalpy -*∆G*^*sp*^(*T*) (in kJ/mol) of the different polar molecules adsorbed at T = 480 K on (**a**) H-β-zeolite, (**b**) 0.25% of rhodium supported by H-β-zeolite, and (c) 2% of rhodium supported by H-β-zeolite (**c**).

The results of the table gives a lot of information to understand the surface physicochemical properties of the various zeolite catalysts. Examples of the values of the specific free enthalpy *∆G*^*sp*^(*T*) of different polar molecules adsorbed on different catalysts are displayed in the Fig. 15. For H-β-zeolite at T = 480 K, the strong amphoteric behavior of this catalyst is emphasized (Fig. 15a). The catalyst actively reacts with the amphoteric solvents (methanol, acetone, tri-CE and tetra-CE), acid (chloroform) and base (ether) molecules.

For 0.25% of rhodium impregnated into H-β-zeolite, similar behavior take place. However, an evolution in the surface acid–base properties of catalyst is observed. The presence of 0.25% of rhodium produces a decrease of the amphoteric character of the catalyst. The magnitudes of methanol and acetone *∆G*^*sp*^ decrease from 10.9 kJ/mol and 14.5 kJ/mol, respectively, to 9.3 kJ/mol and 11.6 kJ/mol. However, there is an increase in the acid character with a diminution of basic specific free enthalpy. It seems that the impregnation of the rhodium in H-β-zeolite causes a reduction in base character and an enhancement in the acid force. The tendency of the decrease of the basic character and the increase of acid character becomes more accentuated for greater percentage of impregnated rhodium (2%Rh, see Fig. 15c). The same behaviors are observed at all the temperatures (Table 12).

##### Other thermodynamic measurements

Some other thermodynamic parameters can be calcualted in this study. Experimental results led to determine the differential heat of adsorption *∆H* ^0^ and the standard entropy change of adsorption *∆S* ^0^ of the probe. These parameters can be obtained from relation (4) by using the two following Eqs. (25) and (26):

25 $$\Delta {H}^{0}= -R\frac{\partial \left(ln{V}_{n}\right)}{\partial \left(\frac{1}{T}\right)}$$

26 $$\Delta {S}^{0}= - \left(\frac{\partial \left(RTlnV\right)}{\partial T}\right)$$

**Figure 16:**
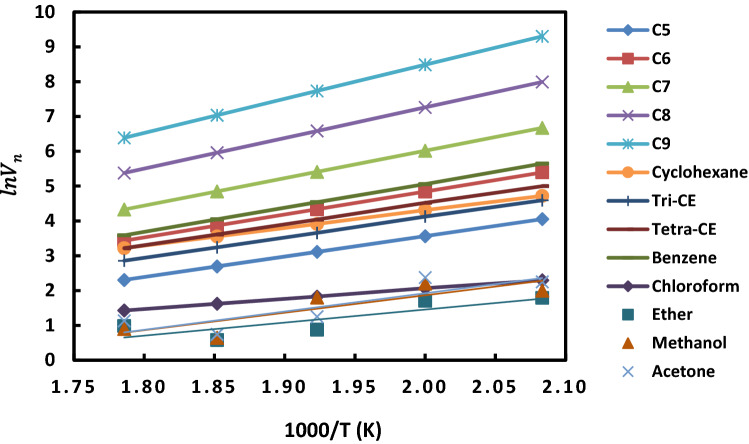
Variations of *lnV*_*n*_ as a function of 1000/T of different polar and n-alkane molecules adsorbed on 2% of rhodium supported by H-β-zeolite.

By plotting $ln{V}_{n}$ as a function of (1/T), one obtained the curves of Fig. 16. A linear dependency was proved and the following general Eq. (27) was obtained for all polar and n-alkanes adsorbed on the catalyst of 2% of rhodium supported by H-β-zeolite:

27 $$ln{V}_{n}=A \left(\frac{1}{T}\right)+B$$

where *A* and *B* are constants depending on the probe nature.

One deduced *∆H* ^0^ and *∆S* ^0^ from Eq. (27):

28 $$\Delta {H}^{0}= -RA;\Delta {S}^{0}= -RB$$

By using relations (25–28) and Fig. 16, we obtained the values of the differential heat the standard entropy change of adsorption given by Table 13.

**Table 13:** Values of $\Delta {H}^{0}(kJ/mol)$, $\Delta {S}^{0}(J {K}^{-1}{ mol}^{-1})$ and the expressions of $\Delta {G}^{0}(T) (kJ/mol)$ of different polar and n-alkane molecules adsorbed on 2% of rhodium supported by H-β-zeolite.

Molecules	$\Delta {H}^{0} (kJ/mol)$	$\Delta {S}^{0} (J {K}^{-1}{ mol}^{-1})$	$\Delta {G}^{0}(T) (kJ/mol)$
C5	− 48.764	− 67.9	− 48.764 + 6.79 × 10^–2^ T
C6	− 54.787	− 69.3	− 54.787 + 6.93 × 10^–2^ T
C7	− 65.456	− 80.9	− 65.456 + 8.09 × 10^–2^ T
C8	− 73.026	− 85.7	− 73.026 + 8.57 × 10^–2^ T
C9	− 81.372	− 92.2	− 81.372 + 9.22 × 10^–2^ T
Cyclohexane	− 42.018	− 48.3	− 42.018 + 4.83 × 10^–2^ T
Tri-CE	− 48.668	− 63.1	− 48.668 + 6.31 × 10^–2^ T
Tetra-CE	− 50.000	− 62.5	− 50.000 + 6.25 × 10^–2^ T
Benzene	− 57.363	− 72.6	57.363 + 7.26 × 10^–2^ T
Chloroform	− 24.401	− 31.7	− 24.401 + 3.17 × 10^–2^ T
Ether	− 31.179	− 50.3	− 31.179 + 5.03 × 10^–2^ T
Methanol	− 41.654	− 67.8	− 41.654 + 6.78 × 10^–2^ T
Acetone	− 43.644	− 71.3	− 43.644 + 7.13 × 10^–2^ T

The values of $\left(-\Delta {H}^{0}\right)$ and $\left(-\Delta {S}^{0}\right)$ of the probe increase when the carbon atom number ${n}_{C}$ increases. Linear relations (29) and (30) were obtained as a function of ${n}_{C}$ for n-alkanes:

29 $$\left(-\Delta {H}^{0}\right)(kJ/mol)= -8.346 {n}_{C}-6.262$$

30 $$\left(-\Delta {S}^{0}\right) (J {K}^{-1}{ mol}^{-1})= -6.50 {n}_{C}-33.71$$

This increase is due to the increase in the boiling points of *n*-alkanes and to the stronger interaction between the solute and catalyst surface.

Table 13 clearly showed that benzene exhibits more negative $\Delta {H}^{0}$ than the corresponding values for n-alkanes with the same carbon atom number (as for example n-hexane or cyclohexane where ${n}_{C}=6$) The more negative the heat, the greater the interaction between the adsorbate and the adsorbent. This can be explained by the specific interactions between benzene’s electrons and the surfaces. The same results were previously observed by Bilgiç and Tümsek^36^.

The $\left(-\Delta {H}^{0}\right)$ values of polar probes increase in the following order for the catalyst 2% of rhodium supported by H-β-zeolite:

Chloroform < Ether < Methanol < Cyclohexane < Acetone < Tri-CE < Tetra-CE < benzene.

This is conform to the relative polarities of polar molecules that decrease in the same order.

#### Variations of the specific enthalpy and entropy of adsorption on different catalysts

From the Table 12 it can be deduced that the curves of *∆G*^*sp*^*(T)* of the polar molecules as a function of the temperature follow linear dependency for all used catalysts in agreement with Eq. (17):

31 $$ \Delta G^{sp} \left( T \right)\, = \,\Delta H^{sp} - T\Delta S^{sp} $$

**Figure 17:**
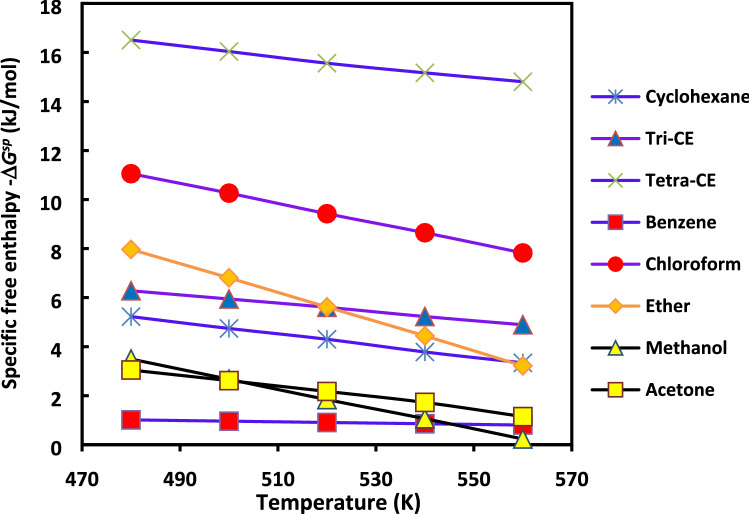
Variations of − *∆G*^*sp*^*(T)* of polar molecules as a function of the temperature in the case of 1.75% impregnated rhodium in H-β-zeolite for a range temperature [480 K, 560 K].

An example of straight lines obtained with the catalyst containing 1.75% of rhodium is shown in Fig. 17.

The specific enthalpy *∆H*^*sp*^ and entropy *∆S*^*sp*^ of adsorption can be calculated by applying Eq. (17) to the data of Table 12. The results are reported in Tables 14 and 15. Note also that all linear regression coefficients, *r*^2^, are close to 1.

**Table 14:** Values of the specific free enthalpy − *∆H*^*sp*^ (kJ/mol) of adsorption of different polar molecules on catalysts as a function of the impregnated rhodium percentage.

%Rh probes	0	0.25	0.5	0.75	1	1.25	1.5	1.75	2
Cyclohexane	9.713	8.788	6.408	5.562	5.614	7.871	14.712	16.581	17.196
Tri-CE	16.959	17.329	18.776	20.903	14.328	19.447	15.694	14.661	14.499
Tetra-CE	32.242	30.634	28.035	25.801	20.477	26.716	26.700	26.716	27.178
Benzene	1.457	1.633	1.539	1.456	0.647	1.907	2.088	2.279	2.377
Chloroform	22.592	27.243	31.054	29.689	4.974	37.111	31.191	30.452	30.654
Ether	54.982	51.848	48.892	46.106	43.478	40.999	38.662	36.459	34.38
Methanol	71.74	60.979	51.832	44.057	37.449	31.831	27.057	22.998	19.548
Acetone	68.321	54.657	43.726	34.981	27.984	22.388	17.91	14.328	11.462

**Table 15:** Values of the specific entropy − *∆S*^*sp*^ (JK^-1^ mol^-1^) of adsorption of different polar molecules on catalysts as a function of the impregnated rhodium percentage.

%Rh probes	0	0.25	0.5	0.75	1	1.25	1.5	1.75	2
Cyclohexane	− 10	− 7	− 2	0	− 1	− 6	− 20	− 24	− 25
Tri-CE	− 19	− 21	− 24	− 34	− 25	− 32	− 20	− 17	− 17
Tetra-CE	− 30	− 27	− 28	− 36	− 31	− 39	− 22	− 21	− 22
Benzene	− 2	− 2	− 2	− 2	− 1	0	− 1	− 3	3
Chloroform	− 30	− 36	− 40	− 38	− 61	− 54	− 42	− 40	− 41
Ether	− 90	− 84	− 80	− 75	− 71	− 67	− 63	− 59	− 56
Methanol	− 127	− 108	− 92	− 78	− 66	− 56	− 48	− 41	− 35
Acetone	− 112	− 89	− 72	− 57	− 46	− 37	− 29	− 23	− 19

The specific enthalpy of interaction between the catalysts and polar molecules is very large for the amphoteric probes as acetone and methanol and for base and acid solvents as ether and chloroform (Table 14). The negative value of the specific entropy of interaction proves the more ordered systems for basic and acidic interactions. This confirms the previous results concerning the acid–base properties of the catalysts.

#### Lewis acid base constants of catalysts

**Figure 18:**
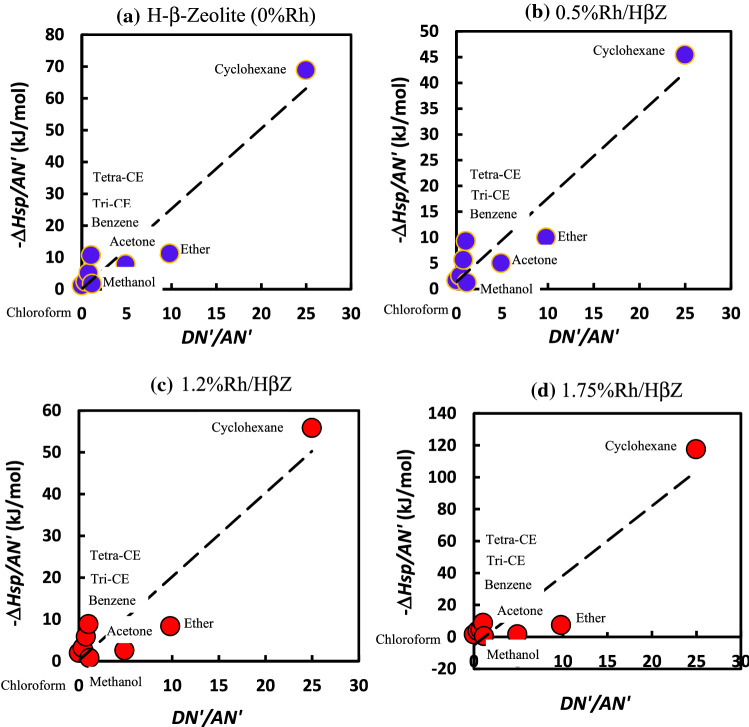
Evolution of − ∆*H*^*sp*^*/AN'* (kJ/mol) versus of DN*'/AN'* of polar molecules adsorbed on different percentages of rhodium impregnated surfaces: (**a**) H-β-Zeolite (0%Rh), (**b**) 0.5%Rh/HβZ, (**c**) 1.25%Rh/HβZ, and (**d**) 1.75%Rh/HβZ (d).

The acid–base constants *K*_*A*_ and *K*_*D*_ of the various catalysts can be obtained using the experimental data and applying the relation (19). To this aim, the evolution of *− ∆Hsp/AN'* as a function of *DN'/AN'* for H-β-zeolite is followed for various rhodium percentages. The Fig. 18 gives examples of these variations, for four amounts of Rh. The extracted acid and base constants obtained for the different solid substrates are presented in Table 16 with the corresponding linear regression coefficients used to fit the linear curves.

**Table 16:** Values of *K*_*D*_, *K*_*A,*_ of different catalysts as a function of the rhodium percentage impregnated in zeolite.

%Rh/HβZ	Acid constant KA (kJ/mol)	Base constant KD (kJ/mol)	Linear regression coefficient R^2^
0	2.522	0.088	0.9117
0.25	2.2691	0.908	0.9076
0.5	1.628	1.365	0.9089
0.75	1.4	1.581	0.9009
1	1.471	0.255	0.9219
1.25	2.007	0.143	0.8777
1.5	3.86	-4.031	0.8689
1.75	4.362	-5.192	0.8643
2	4.5223	-5.58	0.8608

**Figure 19:**
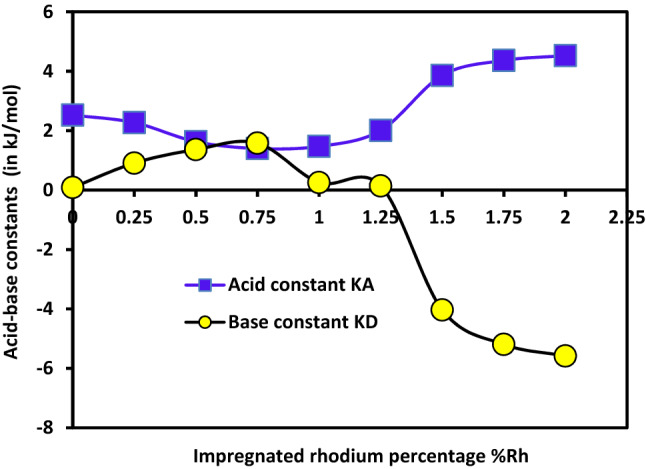
Variations of the acid base constants *K*_*A*_ and *K*_*D*_ (in kJ/mol) of different catalysts as a function of the rhodium percentage impregnated in zeolite by using the classical model.

It seems also interesting to follow the acid and base constants (*K*_*D*_ and *K*_*A*_) as a function of the percentage of rhodium impregnated. The results are given in Fig. 19.

The acid base properties of the zeolite surface are significantly affected by the impregnation of rhodium metal in H-β-zeolite. For a rhodium percentage less than 0.75%, the surface acidity of the catalysts decreases whereas the basicity increases. Conversely, for %Rh larger than 0.75%Rh, an opposite trend takes place since an increase of the acidity and decrease of the basicity are visible. For rhodium percentage larger than or equal to 1.5%Rh, *K*_*D*_ and *K*_*A*_ do not vary with the rhodium percentage. Note that, negative values of the basic constant for rhodium percentages larger than 1.25%Rh are observed. In this range of %Rh, the linear regression coefficients are not very satisfactory since *r*^2^ are comprised between 0.800 and 0.900. Actually, for all the rhodium percentages %Rh, no perfect straight line is obtained. This confirms that the model (Eq. (19)) does not satisfactorily apply to the results. One of reasons for obtaining bad linear regression coefficients r^2^ was the larger value of the ratio DN/AN equal to 25 for cyclohexane, the second reason was the insufficiency of the classical equation to describe with accuracy the experimental results. It becomes then pertinent to employ the Hamieh’s model in order to improve the accuracy of the acid–base constants.

#### Discussion on the light of the new model

**Figure 20:**
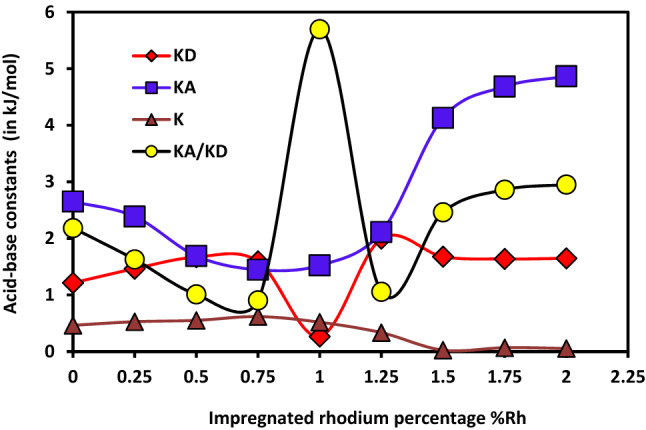
Variations of the acid base constants *K*_*A*_, *K*_*D*_ and *K* (in kJ/mol) and *K*_*A*_/*K*_*D*_ of different catalysts as a function of the impregnated rhodium percentage by using Hamieh’s model.

Some similar irregularities when using Eq. (19) were observed by Hamieh et al.^18,19^. They proposed a new relationship by adding a third parameter *K* reflecting the amphoteric character of solid surfaces. This method is applied here and the Eq. (21) is used to calculate the three acid–base constants *K*_*A*_*, K*_*D*_ and *K* of the various catalysts. These constants are obtained with an excellent three-dimension linear regression coefficients approaching *r*^2^ ≈ 1.000. The obtained results are presented in Table 17 and Fig. 20 where the acid–base constants *K*_*D*_, *K*_*A*_*, K* and the ratio *K*_*A*_*/K*_*D*_ of different substrates are expressed for various rhodium percentages %Rh.

**Table 17:** Acid–base constants *K*_*D*_, *K*_*A*_*, K* and of the ratio *K*_*A*_*/K*_*D*_ of different catalysts as a function of the rhodium percentage %Rh.

%Rh/HbZ	K_D_	K_A_	K	K_A_/K_D_
0	1.215	2.652	0.464	2.18
0.25	1.465	2.387	0.528	1.63
0.5	1.670	1.690	0.552	1.01
0.75	1.596	1.446	0.619	0.91
1	0.267	1.524	0.519	5.70
1.25	1.995	2.112	0.335	1.06
1.5	1.677	4.130	0.026	2.46
1.75	1.637	4.684	0.068	2.86
2	1.648	4.866	0.056	2.95

The H-β-zeolite is more acidic than basic. In the presence of rhodium, the acidity constant *K*_*A*_ decreases from 2.7 to 1.5 kJ/mol when the percentage %Rh increases from 0 to 0.75%. On the opposite, the basicity constant *K*_*D*_, increases from 1.2 to 1.7 kJ/mol and dramatically decreases until 0.3 kJ/mol at rhodium percentage equal to 1%. For Rh percentages larger than 1%Rh, the acid base constants increase until %Rh reaches 1.5% and then stabilize. On the other hand, the amphoteric constant K remains constant up to 1%Rh. It then decreases to reach a plateau above 1.5%Rh. The ratio *K*_*A*_/*K*_*D*_ showing a maximum at 1%Rh confirms the previous results on the incorporation of rhodium into the channels of H-β-zeolite observed when discussing the variations of *RTlnVn*, *∆G*^*sp*^ and the dispersive component of the surface energy ${\gamma }_{s}^{d}$ of the different catalysts.

It seems interesting to compare the order of magnitudes of the constants with those reported in the literature. Bilgiç and Tümsek determined the surface acid base properties of MgY and NH4Y using inverse gas chromatography^36^. According to results obtained by the above authors for *K*A and *K*D, the surface of MgY exhibits predominantly basic character with the ratio of *K*D/*K*A = 3.50, while surface of NH4Y shows a less basic character with the ratio of *K*D/*K*A = 2.61. These results showed basic than acidic character of the zeolite materials. However, when comparing these data with those obtained in our study, it appears that our catalysts are rather acidic than basic since the ratios KA/*K*D are comprised between 0.9 and 5.7. The difference between the two materials results from the presence of framework oxygens adjacent to alkali cations which are the Lewis basic sites in zeolites. This was previously proved by Bilgic and Tumsek^36^, Barr and Lishka^37^, Okamoto et al.^38^ and Vinek et al.^39^. Other catalysts exhibit acidic surface similar to the catalysts of the present study. As an example, the sepiolite surface characterized by Morales et al.^40^ for which the ratio of acid base constants KA/*K*D was equal to 3.

It seems also relevant to evaluate the error committed on the values of acid base constants. To this aim, the following approach is employed.

The error committed on the net retention time is:

$${{10}^{-3}min\le \Delta t}_{n}\left(probe\right)\le 3\times {10}^{-3}min$$

The relative standard deviation on the retention time is given by the following inequalities:

$$5\times {10}^{-5} \le \frac{\Delta {t}_{n}\left(probe\right)}{{t}_{n}\left(probe\right)}\le {10}^{-4}$$

This gives a relative standard deviation on the net retention volume:

$$5\times {10}^{-5} \le \frac{\Delta {V}_{n}\left(probe\right)}{{V}_{n}\left(probe\right)}\le {10}^{-4}$$

And therefore, we obtain for free enthalpy of adsorption the following error:

$$5\times {10}^{-4}kJ/mol \le \Delta \left({\Delta G}_{a}^{0}\right)\le 3\times {10}^{-3}kJ/mol$$

Moreover, the relative deviation is given by:

$$3\times {10}^{-4} \le \frac{\Delta \left({\Delta G}_{a}^{0}\right)}{{\Delta G}_{a}^{0}}\le 5\times {10}^{-4}$$

And the error on the specific free enthalpy reads as:

$${10}^{-3}kJ/mol \le \Delta \left(\Delta {G}_{a}^{sp}\right)\le 6\times {10}^{-3}kJ/mol$$

Finally, the relative error committed on the acid–base constants *K*_*A*_, *K*_*B*_ and *K* are:

$$1\times {10}^{-3} \le \frac{\Delta \left({K}_{A, B}\right)}{{K}_{A, B}}\le 2\times {10}^{-3}$$

Therefore, the error committed on the values of acid base constants is equal to $5\times {10}^{-3}$.

## Conclusion

In this paper, new thermodynamic methods and models were developed to study the surface energy and acid base properties of H-β-zeolite impregnated with rhodium metal at different percentages %Rh. The effect of the temperature and the rhodium content on the acid base properties in Lewis terms of the various catalysts were analyzed by inverse gas chromatography at infinite dilution. The variation of *RTlnVn* of n-alkanes adsorbed on the catalysts with the rhodium percentage revealed the presence of a maximum at %Rh = 0.75%. Conversely, for polar solvents the maximum occurs at 1.00%Rh. This is due to the variation of some surface groups because of the change in the acid base properties of the catalysts when adding rhodium in zeolite.

The specific surface area *S*_*BET*_ of different catalysts decreases with the rhodium percentage until 0.5%Rh, followed by a slight increase to reach a local maximum at 1%Rh. Finally, *S*_*BET*_ decreases up to a plateau observed for %Rh larger than 1.50%. The same conclusion was observed for the microporous volume. The highest value of the specific surface area was obtained for H-β-zeolite. In the presence of rhodium, the metal particles block the micropores causing a decrease in the specific surface area and in the catalyst microporosity. However, the increase of the specific surface area, for the catalysts containing a rhodium percentage comprised between 0.5 and 1.0, can result from the smaller particle sizes that cannot block the zeolite micropores. For catalyst with a rhodium percentage larger than 1.50% Rh, the much lower observed surface area and pore volume is certainly due to the larger nanoparticles which block the micropores. The same behavior was observed when studying the variations $\frac{{d\gamma }_{s}^{d}}{dT}$ of catalysts as a function of the impregnated rhodium percentage regardless of the molecular model of n-alkane surface areas used.

The results relative to the specific free enthalpy *∆G*^*sp*^(*T*) of different polar molecules adsorbed on H-β-zeolite clearly demonstrated the strong amphoteric behavior of all supported Rh catalysts. The rhodium supported by H-β-zeolite actively react with the amphoteric solvents (methanol, acetone, tri-CE and tetra-CE), acid (chloroform) and base (ether) molecules. A decrease of the amphoteric character of the catalyst with 0.25% of rhodium is reported. The magnitudes of methanol and acetone *∆G*^*sp*^ decrease from 10.9 kJ/mol and 14.5 kJ/mol, respectively, to 9.3 kJ/mol and 11.6 kJ/mol. Whereas, an increase in the acid character with a decrease of basic specific free enthalpy were highlighted. It seems that the impregnation of the rhodium in H-β-zeolite causes a decrease in base character and an increase in the acid magnitude. The tendency of the decrease of basic character and the increase of acid character became more accentuated for greater percentage of impregnated rhodium (2%Rh) for all temperatures.

The classic Gutmann relationship was not well suited for an accurate determination of the acid base constants. Negative values of the basic constant for rhodium percentage more than 1.25%Rh coupled to weak linear regression coefficients of the order of 0.8 and 0.9 are obtained. The previous results were corrected by applying the Hamieh’s model. In this case, the acid–base constants *K*_*A*_*, K*_*D*_ and *K* of the various catalysts were determined with an excellent accuracy. The H-β-zeolite is more acidic than basic with more important specific interactions. The acidity constant *K*_*A*_ decreases with the Rh content while the basicity constant *K*_*D*_, increases up to 1%Rh. At the same time, the amphoteric constant K remains constant until 1%Rh and then decreases to reach its plateau from 1.5%Rh. An interesting correlation was highlighted between the surface specific area of the various catalysts, the rhodium percentage in zeolites and the specific acid base interactions between the catalysts and the polar organic molecules.

## References

[1] Chow AHL, Tong HHY, Shekunov BY, York P (2004). Letter to editor: Use of inverse gas chromatography (IGC) to determine the surface energy and surface area of powdered materials. Pharma. Res..

[2] Askin A, Yazici DT (2008). A study of the surface analysis of some water-soluble polymers by inverse gas chromatography. Surf. Interface Anal..

[3] Sreekanth TVM, Reddy KS (2007). Analysis of solvent-solvent interactions in mixed isosteric solvents by inverse gas chromatography. Chromatographia.

[4] Yang YC, Yoon PR (2007). Examination of the surface properties of kaolinites by inverse gas chromatography: Acid–base properties. Korean J. Chem. Eng..

[5] Ansari DM, Price GJ (2004). Polymer, chromatographic estimation of filler surface energies and correlation with photodegradation of kaolin filled polyethylene. Polymer.

[6] Fekete E, Móczo J, Pukánszky B (2004). Determination of the surface characteristics of particulate fillers by inverse gas chromatography at infinite dilution: A critical approach. J. Colloid Interface Sci..

[7] Ward, T. C., Lloyd, D. R., Schreiber, H. P. (Eds.), Inverse Gas Chromatography, ACS Symp. Ser. No. **391** (Washington, DC, 1989).

[8] Cline D, Dalby R (2002). Predicting the quality of powders for inhalation from surface energy and area. Pharm. Res..

[9] Santos JMRCA, Guthrie JT (2005). Analysis of interactions in multicomponent polymeric systems: The key-role of inverse gas chromatography. Mater. Sci. Eng. R..

[10] Gamelas JAF (2013). The surface properties of cellulose and lignocellulosic materials assessed by inverse gas chromatography: A review. Cellulose.

[11] Mukhopadhyay P, Schreiber HP (1995). Aspects of acid–base interactions and use of inverse gas chromatography. Colloids Surf. A.

[12] Gamelas JAF, Ferraz E, Rocha F (2014). An insight into the surface properties of calcined kaolinitic clays: The grinding effect. Colloids Surf. A.

[13] Feeley JC, York P, Sumby BS, Dicks H (1998). Processing effects on the surface properties of α-lactose monohydrate assessed by inverse gas chromatography (IGC). Int. J. Pharm..

[14] Ticehurst, M.D. Characterisation of the surface energetics of pharmaceutical powders by inverse gas chromatography. University of Bradford, York, Ph.D. thesis, 1995.

[15] Voelkel A, Grzeskowiak T (2000). The use of solubility parameters in characterization of titanate modified silica gel by inverse gas chromatography. Chromatographia.

[16] Newell E, Buckton G, Butler DA, Thielmann F, Williams DR (2001). The use of inverse phase gas chromatography to measure the surface energy of crystalline, amorphous, and recently milled lactose. Pharm. Res..

[17] Kalantzopoulou FR, Artemiacti T, Bassiotis I, Katsanos NA, Plagianakos V (2001). Time separation of adsorption sites on heterogeneous surfaces by inverse gas chromatography. Chromatographia.

[18] Hamieh T, Schultz J (2002). New approach to characterise physicochemical properties of solid substrates by inverse gas chromatography at infinite dilution. I. Some new methods to determine the surface areas of some molecules adsorbed on solid surfaces. J. Chromatogr. A..

[19] Hamieh T, Rageul-Lescouet M, Nardin M, Haidara H, Schultz J (1997). Study of acid–base interactions between some metallic oxides and model organic molecules. Colloids Surf. A Phys. Eng. Aspects..

[20] Nakatsuji T, Komppa V (2002). A catalytic NO_*x*_ reduction system using periodic steps, lean and rich operations. Catal. Today.

[21] Carroll AM, O’Sullivan TP, Guiry PJ (2005). The development of the asymmetric rhodium-catalysed olefin hydroboration. Adv. Synth. Catal..

[22] Matolin V, Masek K, Elyakhloufi MH, Gillet E (1993). Adsorption of CO on small supported rhodium particles: SSIMS and TPD study. J. Catal..

[23] Hwang CP, Yeh CT, Zhu QM (1999). Rhodium-oxide species formed on progressive oxidation of rhodium clusters dispersed on alumina. Catal. Today.

[24] Navio JA, Colon G, Litter MI, Bianco GN (1996). J. Mol. Catal. A: Chem..

[25] Zhang X, Qian L, Xu P, He H, Du Q (2008). Study of H-β–zeolite supported Rh catalyst by inverse gas chromatography. Chem. Eng. J..

[26] Moloy EC, Davila LP, Shackelford JF, Navrotsky A (2002). A relationship between energetics and internal surface area. Micropor. Mesopor. Mater..

[27] Saint Flour C, Papirer E (1982). Gas-solid chromatography. A method of measuring surface free energy characteristics of short glass fibers. 1. Through adsorption isotherms. Ind. Eng. Chem. Prod. Res. Dev..

[28] Saint Flour C, Papirer E (1982). Gas-solid chromatography: method of measuring surface free energy characteristics of short fibers. 2. Through retention volumes measured near zero surface coverage. Ind. Eng. Chem. Prod. Res. Dev..

[29] Papirer E, Brendlé E, Balard H, Ozil F (1999). IGC determination of surface properties of fullerenes: Comparison with other carbon materials. Carbon.

[30] Conder JR, Young CL (1979). Physical Measurements by Gas Chromatography.

[31] Dorris GM, Gray DG (1980). Adsorption of normal-alkanes at zero surface coverage on cellulose paper and wood fibers. J. Colloid Interface Sci..

[32] Fowkes FM, Andrade JD (1985). Surface and Interfacial Aspects of Biomedical Polymers.

[33] Gutmann V (1978). The Donor–Acceptor Approach to Molecular Interactions.

[34] Riddle FL, Fowkes FM (1990). Spectral shifts in acid-base chemistry Van der Waals contributions to acceptor numbers, Spectral shifts in acid–base chemistry. 1. van der Waals contributions to acceptor numbers. J. Am. Chem. Soc..

[35] Hamieh T (2020). Study of the temperature effect on the surface area of model organic molecules, the dispersive surface energy and the surface properties of solids by inverse gas chromatography. J. Chromatogr. A.

[36] Bilgiç C, Tümsek F (2007). Determination of the acid/base properties of MgY and NH4Y molecular sieves by inverse gas chromatography. J. Chromatogr. A.

[37] Barr TL, Lishka MA (1986). ESCA studies of the surface chemistry of zeolites. J. Am. Chem. Soc..

[38] Okamoto Y, Maezawa M, Imanaka T (1988). Electronic structure of zeolites studied by X-ray photoelectron spectroscopy. J. Catal..

[39] Vinek H, Noller H, Ebel M, Schwarz K (1977). X-ray photoelectron spectroscopy and heterogeneous catalysis, with elimination reactions as an example. J. Chem. Soc. Faraday Trans..

[40] Morales E, Dabrio MV, Herrero CR, Acosta L (1991). Acid/base characterization of sepiolite by inverse gas chromatography. Chromatographia.

